# Intra‐articular injection of rapamycin microparticles prevent senescence and effectively treat osteoarthritis

**DOI:** 10.1002/btm2.10298

**Published:** 2022-05-05

**Authors:** Kaamini M. Dhanabalan, Ameya A. Dravid, Smriti Agarwal, Ramanath K. Sharath, Ashok Kumar Padmanabhan, Rachit Agarwal

**Affiliations:** ^1^ Centre for BioSystems Science and Engineering Indian Institute of Science Bengaluru India; ^2^ Department of Orthopaedics MS Ramaiah Medical College Bengaluru India

**Keywords:** autophagy, intra‐articular therapy, microparticles, osteoarthritis, PLGA, rapamycin, senescence

## Abstract

Trauma to the knee joint is associated with significant cartilage degeneration and erosion of subchondral bone, which eventually leads to osteoarthritis (OA), resulting in substantial morbidity and healthcare burden. With no disease‐modifying drugs in clinics, the current standard of care focuses on symptomatic relief and viscosupplementation. Modulation of autophagy and targeting senescence pathways are emerging as potential treatment strategies. Rapamycin has shown promise in OA disease amelioration by autophagy upregulation, yet its clinical use is hindered by difficulties in achieving therapeutic concentrations, necessitating multiple weekly injections. Rapamycin‐loaded in poly(lactic‐co‐glycolic acid) microparticles (RMPs) induced autophagy, prevented senescence, and sustained sulphated glycosaminoglycans production in primary human articular chondrocytes from OA patients. RMPs were potent, nontoxic, and exhibited high retention time (up to 35 days) in mice joints. Intra‐articular delivery of RMPs effectively mitigated cartilage damage and inflammation in surgery‐induced OA when administered as a prophylactic or therapeutic regimen. Together, the study demonstrates the feasibility of using RMPs as a potential clinically translatable therapy to prevent the progression of post‐traumatic OA.

## 1 INTRODUCTION

Osteoarthritis (OA), a chronic progressive joint disorder, has affected nearly 500 million people worldwide.^1^ As a progressive degenerative disease, cartilage damage can lead to synovitis, osteophyte formation, and hypertrophy of the entire joint capsule, ultimately leading to loss of function.^2^ Acute damage to the cartilage can occur with trauma to the joint such as vehicle accidents, falls, sports injuries, and military activities. Chronic damage occurs with aging, coupled with obesity, diabetes, and hormonal imbalances.^2^ The current treatment strategies, such as nonsteroidal anti‐inflammatory drugs, steroids, local viscosupplementation, and physiotherapy, address only the symptoms like pain and inflammation.^3^, ^4^ There is a lack of any disease‐modifying drug at the clinics to prevent, halt, or reverse the disease progression.^5^

Growing evidence points at deficient nutrient recycling mechanisms during stress conditions (mechanical, obesity, aging, injuries, etc.) and an imbalance of the catabolic and anabolic pathways as the primary etiology of OA.^6^, ^7^, ^8^, ^9^, ^10^, ^11^ Autophagy is a cellular homeostasis mechanism that maintains the balance between catabolic and anabolic pathways, and autophagic imbalance in chondrocytes is widely implicated in the onset and progress of OA.^12^, ^13^, ^14^, ^15^, ^16^ Failure in autophagy upregulation during these stress conditions can make the cells apoptotic, leading to diminished repair and remodeling capability in cartilage.^12^, ^13^, ^17^, ^18^, ^19^

Chondrocytes under constant oxidative or genotoxic stress conditions can also turn senescent, leading to the secretion of inflammatory cytokines.^20^, ^21^, ^22^ The senescent chondrocytes secrete a host of inflammatory proteins such as IL‐6, IL‐8, and IL‐17, together known as senescent‐associated secretory phenotype (SASP) factors, which attracts immune cells leading to chronic inflammation.^23^, ^24^ Selective modulation of senescent cells has been one of the widely sought‐after OA disease‐modifying strategies, where a wide variety of senolytic or senomorphic drugs are being evaluated, with a few of them undergoing clinical trials.^25^, ^26^, ^27^, ^28^, ^29^ Together, these studies suggest that autophagy activation and senescence modulation are critical for maintaining homeostasis of the articular cartilage, and targeting these mechanisms can act as novel treatments to modify OA outcomes.^26^, ^27^, ^29^, ^30^, ^31^, ^32^

Rapamycin, a well‐known immune modulator and an antibiotic used routinely in clinics for various diseases, has been shown to delay OA progression in mice models.^30^, ^33^ Rapamycin induces autophagy via mTOR inhibition, pushes the cells from stress into the nutrient survival pathway, and restores the chondrocyte's health.^30^, ^33^, ^34^ Rapamycin also prevents senescence and stalls the production of SASP factors.^35^, ^36^, ^37^, ^38^, ^39^ It is hence evident that rapamycin is a promising drug for OA treatment. However, free drug administration via intra‐articular (IA) injection is challenging due to effective lymphatic clearance in joints and hence necessitates high dose administration and frequent injections. Compounds with a molecular weight close to rapamycin (914 g mol^−1^) such as Evans blue (963 g mol^−1^) and Ceftazidime (564 g mol^−1^) exhibit IA residence time of less than 1 h.^40^ Small molecules (<10 kDa) such as corticosteroids frequently administered intra‐articularly for pain management of OA have residence time only up to 1–4 h.^41^ Repeated injections to sustain therapeutic effect can lead to pain, joint irritations and infections, and poor clinical acceptance.

Studies that have attempted to deliver OA disease‐modifying agents such as inhibitors of catabolic enzymes and cytokine receptors via IA injections have met with little clinical success.^42^, ^43^, ^44^ One of the main reasons for the lack of success of these potential disease‐modifying agents is an insufficient therapeutic concentration for prolonged duration in the joint, owing to the efficient and rapid lymphatic clearance inside the knee joint.^41^ Senolytic drug, UBX0101, which was promising in treating OA in mice,^25^ recently failed to show efficacy after 12 weeks of a single IA injection (maximum dose of 4 mg per patient) in phase II clinical trial (NCT04129944). It is hypothesized that the lack of efficacy of UBX0101 was due to its rapid clearance from the joints. Another phase I clinical trial using two injections (4 mg each) at weeks 0 and 4 is underway (NCT04229225). Thus, it becomes evident that sufficient and prolonged therapeutic concentration inside the articular joints necessitates several frequent administrations, which significantly reduces patient compliance at clinics.

IA drug delivery systems using polymer‐based slow‐release formulation can serve as the key in bringing many potent drugs into clinics.^45^, ^46^ Poly lactic‐co‐glycolic acid‐based system (PLGA) is a robust and widely used clinical drug delivery system.^46^, ^47^ We previously showed that PLGA‐based drug delivery systems have prolonged retention time in the murine knee joint.^37^ Therefore, we hypothesized that IA injections of rapamycin in PLGA‐based microparticles (RMPs) could prolong the drug's residence time and could be used to treat OA.

We report that the rapamycin PLGA microparticles induce autophagy and prevent senescence in primary human articular chondrocytes (HACs) obtained from OA patients. The formulation sustained the production of sGAG production in stressed micromass cultures. When administered as prophylactic or therapeutic regimens, the rapamycin particle formulation ameliorates surgery‐induced OA in mice. To our knowledge, this is the first report of successful mice OA therapy using rapamycin in an injectable microparticle formulation, and such strategies should be explored further for translation to humans.

## 2 RESULTS

### 2.1 Chondrocytes from OA patients exhibited high senescence and diminished autophagy near OA lesions

**FIGURE 1:**
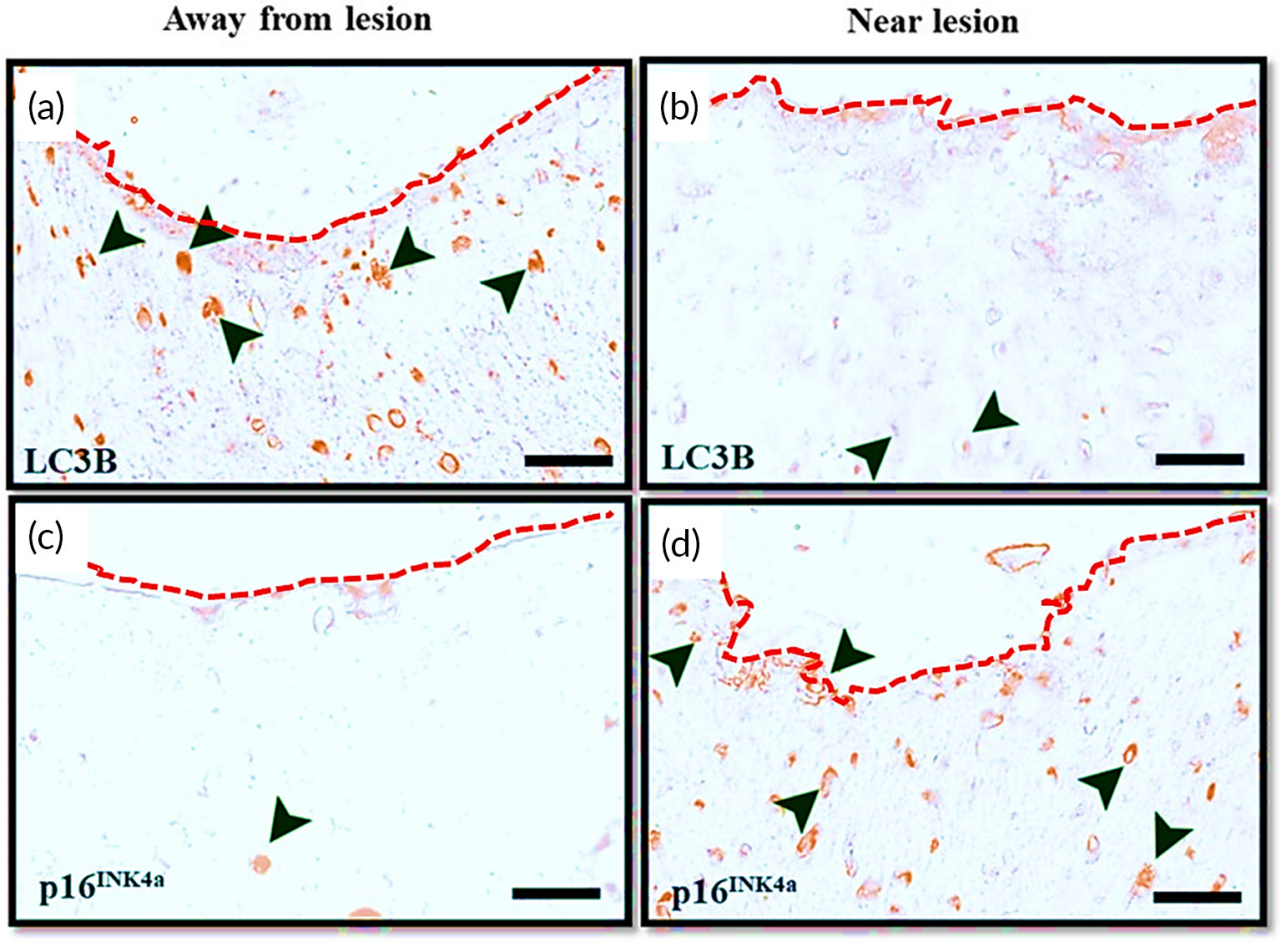
Chondrocytes from human osteoarthritic cartilage bear a higher senescence burden and diminished autophagy near lesions. Immunohistochemical staining of LC3B in human osteoarthritic cartilage sections obtained from regions (a) away from lesion and (b) near osteoarthritis (OA) lesion in knee joint cartilage. Immunohistochemical staining of p16^INK4a^ in human osteoarthritic cartilage sections obtained from regions (c) away from lesion and (d) near OA lesion in knee joint cartilage. Black arrows indicate the cells positive for the respective markers. The red dotted line represents the articular cartilage surface. The images represent data collected from three OA patients who underwent a total knee replacement. Scale bar = 25 μm

To determine the basal autophagy and senescence burden of articular chondrocytes in end‐stage OA disease of humans, we examined the sections from explanted cartilage obtained from patients undergoing knee replacement surgery. Immunohistochemistry was used for respective staining markers of autophagy (LC3BII [microtubule‐associated protein 1B‐Light chain 3]) and senescence (p16^INK4a^ [cyclin‐dependent kinase inhibitor 2A]). Knee joints from OA patients undergoing total knee arthroplasty were obtained with appropriate consents and approvals. The tibial articulating surface was examined for gross morphology, and the lesioned areas with evident subchondral bone erosion were identified and labeled as OA‐lesioned areas. Areas bearing healthy smooth cartilage surface morphology were identified as nonlesioned areas (Figure S1). The nonlesioned areas exhibited higher LC3B‐positive cells and comparatively fewer p16^INK4a^‐positive cells suggesting the cartilage was healthy with active basal autophagy and fewer senescent cells (Figure 1a,c). However, the chondrocytes near the OA lesioned surface had lesser LC3B and high p16^INK4a^‐positive cells, indicating that the cells near the OA lesioned cartilage exhibited high senescence and diminished autophagy (Figure 1b,d), which is in line with previously published results.^13^, ^48^, ^49^

### 2.2 PLGA microparticles provide a tunable platform to release rapamycin

**FIGURE 2:**
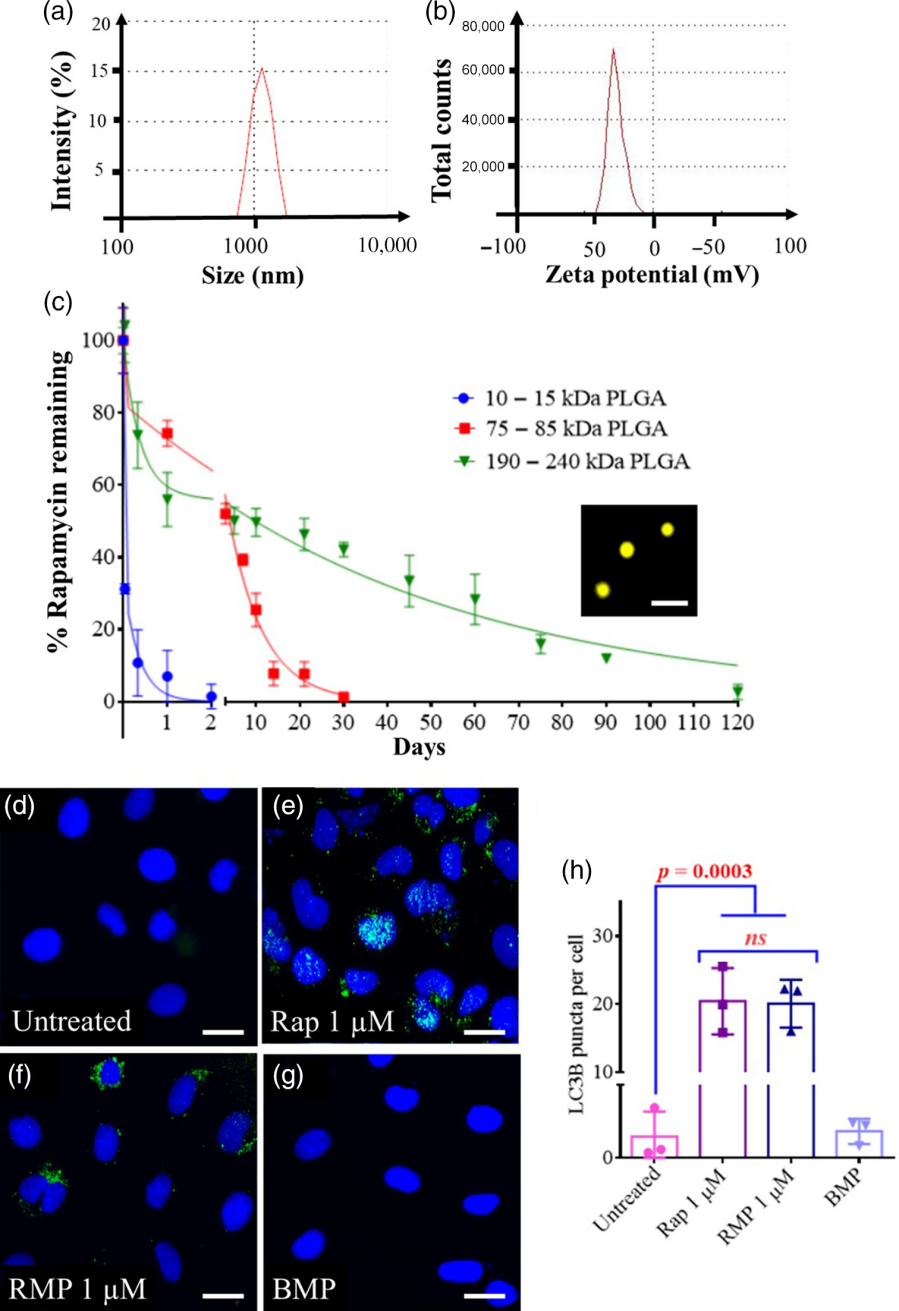
Rapamycin microparticles induce autophagy in primary human chondrocytes. (a) Size distribution of 10–15 kDa poly(lactic‐co‐glycolic acid) (PLGA) particles measured by dynamic light scattering. (b) Zeta potential of 10–15 kDa PLGA particles. (c) In vitro release profiles of rapamycin from microparticles synthesized from different molecular weight PLGA polymers (*n* = 3 per group at each time point). Inset in (c) shows fluorescence microscopy image of Cy3 loaded PLGA particles (molecular weight 10–15 kDa). Scale bar = 3 μm. Fluorescence microscopy images of HACs stained with DAPI and LC3B after (d) no treatment, (e) free rapamycin (1 μM), (f) RMPs; 1 μM rapamycin) and, (g) BMPs treatment. (h) Quantification of LC3B puncta per cell using ImageJ software (n = 3 per group). Plots were representative of data collected from three osteoarthritis (OA) patients. Data in graphs represent the mean ± SD, and *p* values were determined by one‐way analysis of variance (ANOVA) and Tukey's post hoc tests. BMP, blank microparticles; HACs, human articular chondrocytes; *ns*, nonsignificant; RMP, rapamycin‐loaded microparticles. Scale bar = 10 μm

Size plays a vital role in the prolonged joint retention time of PLGA microparticles. We had previously shown that particles in a size range of 1 μm exhibited high residence time in murine^37^ and rodent^50^ knee joints, and hence we proceeded to use this size range for our current study. Dynamic light scattering analysis revealed an average diameter of 1039 ± 188 nm (Figure 2a; Table S1), and the zeta potential was −18 mV due to the presence of acid end‐capped PLGA polymer in the particles (Figure 2b).

To assess the tunability, microparticles of different molecular weights of PLGA were used to encapsulate rapamycin. The size and rapamycin encapsulation efficiency (EE) are listed in Supplementary Table S1. It was observed that the PLGA MPs of molecular weight 10–15 kDa released the drug as quickly as in 48 h, while PLGA MPs of molecular weight 75–85 kDa continued to release the drug up to 45 days. We varied the PLA:PGA ratio of the PLGA and found that the 190–240 kDa PLGA containing PLA:PGA (85:15) could extend the release of the drug for 120 days (Figure 2c). Thus, the particle platform was tunable and allowed the release of rapamycin in a controlled and sustained manner. For 48 h in vitro experiments, the shorter releasing particles (10–15 kDa) were used, and since our animal model experiments were 2 months long, we used the longer releasing particles (75–85 kDa). While 190–240 kDa showed an even more extended‐release profile (~4 months), it was not used in subsequent experiments as our mice experiments were 2 months long. Such extended releasing formulations can be useful for further testing in larger animal models and humans.

### 2.3 RMPs induce autophagy in primary HACs

Rapamycin is a well‐known autophagy inducer that acts by inhibiting the mTOR signaling pathway.^51^ We had previously shown that rapamycin as a free drug and MP formulation induced autophagy in the C28/I2 (human chondrocyte cell line) (Figure S2a–e).^37^ Here, we found that in the primary HACs, both free rapamycin and RMPs successfully induced autophagy as visualized by the LC3B puncta in the cells.

Untreated HACs showed a low diffuse signal with very few puncta indicative of basal autophagy (Figure 2d). In contrast, upon treatment with free rapamycin (1 μM) or RMPs (equivalent rapamycin dose of 1 μM) (Figure 2e,f) for 48 h, the LC3BII signal was observed as punctate signals. The results from RMPs were comparable to that of the free rapamycin‐treated groups (Figure 2e). The LC3B puncta per cell were higher in free rapamycin, and RMP‐treated cells than untreated cells. The untreated cells had an average of fewer than 2 puncta per cell indicative of basal autophagy, while the rapamycin and RMP treated groups exhibited around 25 puncta per cell indicative of upregulated autophagy upon rapamycin treatment (Figure 2h). These findings suggest that RMPs were able to induce autophagy in the HACs.

### 2.4 RMPs prevented senescence in primary HACs

**FIGURE 3:**
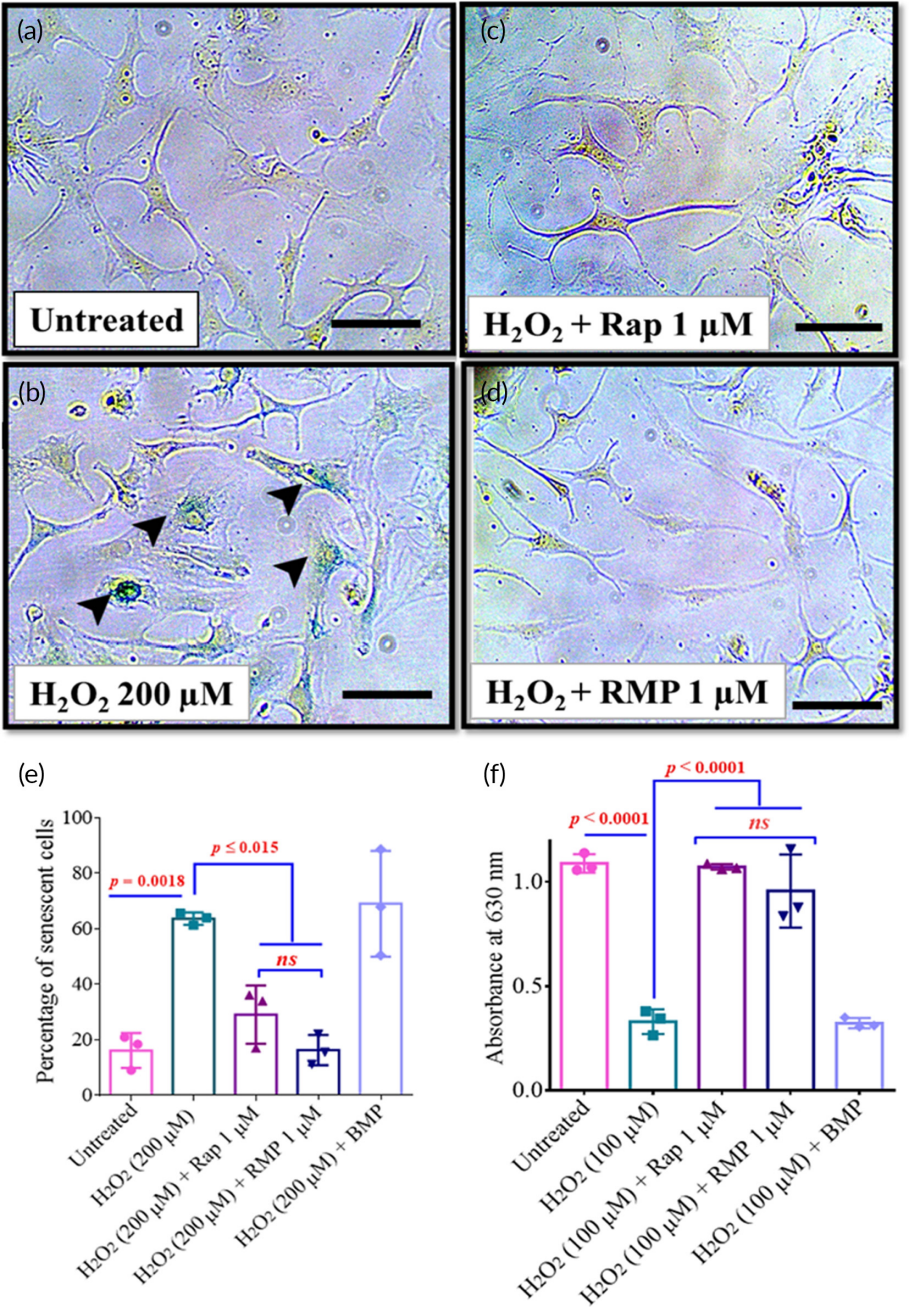
RMPs prevented senescence in primary HACs and sustained sGAG production in micromass cultures exposed to oxidative stress. SA‐β Gal‐stained images of primary HACs exposed to (a) no treatment, (b) oxidative (H_2_O_2_) stress, (c) oxidative (H_2_O_2_) stress with free rapamycin (1 μM), and (d) oxidative (H_2_O_2_) stress with RMPs (1 μM). Scale bar ‐ 40 μm. (e) Percent of senescent HACs after culturing in oxidative (H_2_O_2_) stress condition and different cotreatments (*n* = 3 per group) for 48 h. (f) sGAG production in stressed micromass cultures measured as absorbance of guanidine HCl extracted Alcian blue stain. Images and graphs were representatives of data collected from three osteoarthritis (OA) patients. Data in graphs represent the mean ± SD and *p* values were determined by one‐way analysis of variance (ANOVA) and Tukey's post hoc tests. *p*‐value <0.05 was considered significant. BMPs, blank microparticles; HACs, human articular chondrocytes; *ns*, nonsignificant; RMP, rapamycin‐loaded microparticles. ****p < 0.0001

Chondrocytes in the knee joint get exposed to various stress conditions such as DNA damage and increased ROS production.^52^, ^53^, ^54^ We exposed them to oxidative stress by adding H_2_O_2_ to simulate these stress conditions in the chondrocytes. For the initial dose optimization studies, we treated the chondrocytes (C28/I2) with H_2_O_2_ (100 and 200 μM) to check for the percentage of senescence. We chose 200 μM of H_2_O_2_ as the percentage of senescent cells was statistically not different from the 100 μM of H_2_O_2_ (Figure S3a). Next, we cotreated a few of the experimental groups with free rapamycin and RMPs (1 μM) for 48 h and stained the cells with SA‐β Gal (senescent marker) (Figure 3a–d). HACs when exposed to 200 μM of oxidative stress agent (H_2_O_2_), resulted in 30% of cells becoming senescent, while the cotreatment with 1 μM RMPs brought down the percentage of senescent cells to less than 10%, which was comparable to cells with no oxidative stress (Figure 3e).

Similar results were also obtained with C28/I2 cell lines when cells were treated to genotoxic (BrdU, 200 μM) stress or oxidative (H_2_O_2_, 200 μM) (Figures S4 and S5). Overall, these results indicate that rapamycin in MPs was potent and active post encapsulation and prevented senescence under different stress conditions in HACs and C28/I2 cell lines.

### 2.5 RMPs help to sustain sGAG production in stressed micromass cultures

When seeded at a high density along with the growth factor (TGF‐β), chondrocytes form three‐dimensional (3D) micromasses that exhibit high deposition of extracellular matrix components such as sulfated glycosaminoglycans (sGAG).^55^ They serve as in vitro 3D culture models and are widely used in cartilage research to evaluate various stress conditions and treatment modalities. The micromasses derived from C28/I2 cells were treated with genotoxic or oxidative stress to simulate physiological stress conditions present during knee joint trauma. These were also cotreated with rapamycin as a free drug or MP formulation to evaluate the sGAG production in the stressed micromasses.

The concentrations of BrdU (600 μM) and H_2_O_2_ (100 μM) were obtained by our initial optimization experiments. In the BrdU optimization experiment, two doses of BrdU were chosen: 400 and 600 μM. The higher dose of BrdU (600 μM) reduced the sGAG production by twofold compared to untreated, and hence we continued to use this concentration for our future experiments (Figure S6a). For the H_2_O_2_ dose optimization experiment, we treated the micromasses with 100 and 200 μM of H_2_O_2_. Since there was no statistical difference between 100 and 200 μM of H_2_O_2_, we, therefore, performed all our subsequent experiments with 100 μM H_2_O_2_ (Figure S6b).

The micromasses derived from HACs were treated with oxidative stress using externally added H_2_O_2_ (100 μM) and cotreated with free rapamycin or RMPs. We wanted to evaluate whether rapamycin or RMPs can sustain the production of sGAG in micromasses under oxidative stress conditions. In micromasses derived from HACs, H_2_O_2_ treatment resulted in nearly threefold lower sGAG production than the vehicle‐treated groups, while the free rapamycin or RMPs treatment rescued the sGAG production and was comparable to untreated groups (Figure 3f). Similar results were also obtained with micromasses formed from C28/I2 cells when treated with oxidative (H_2_O_2_, 100 μM) or genotoxic (BrdU, 600 μM) stress for 48 h (Figure S7a,b).

To evaluate the long‐term sGAG production by the stressed micromasses, we treated micromass formed from C28/I2 cells for 8 days with BrdU (600 μM) or H_2_O_2_ (100 μM) along with cotreatment groups containing rapamycin or RMPs at 1 μM dose. In the BrdU (600 μM) treated group, the absorbance values dropped to almost sixfold compared to untreated groups, while the RMP‐treated groups sustained the sGAG production on par with free rapamycin‐treated groups (Figure S7b). Likewise, sGAG production dropped to almost threefold with H_2_O_2_ treatment, and the rapamycin or RMP‐treated group significantly increased the sGAG production at par with untreated groups (Figure S7d). In summary, these results suggest that rapamycin and RMPs were potent anabolic agents and sustained sGAG production in stressed chondrocytes for long durations under oxidative and genotoxic stress.

### 2.6 PLGA MPs of high molecular weight had longer residence time in mice knee joints

To estimate the dosage and frequency of rapamycin to be administered for mice models of OA, we wanted to determine the residence time of rapamycin in the knee joint space. Since in our in vitro rapamycin release data, 75–85 kDa PLGA MPs showed sustained release for more than a month (Figure 2c), we chose this formulation for all subsequent mice experiments. As it is difficult to assess the residence time of nonfluorescent drugs like rapamycin, we assessed the in vitro release rate of a fluorescent molecule, Cy7, which has a comparable molecular weight (Mw) (Mw of Cy7: 626.7 g mol^−1^; Rapamycin: 914.1 g mol^−1^) and hydrophobicity (LogP of Cy7 3.4; LogP of Rapamycin: 4.81) as rapamycin. The in vitro release profile of the RMPs and the Cy7 particles in 1x phosphate‐buffered saline (PBS; Figure S8) followed a similar trend, and a two‐phase decay curve using nonlinear regression (least square method) was used to fit the release pattern of rapamycin and Cy7 dye from PLGA particles. Rapamycin and Cy7 dye followed a typical biphasic release from PLGA MPs, in line with the previously published literature.^37^, ^56^, ^57^

Next, we injected Cy7 containing PLGA MPs intra‐articularly in mice and monitored the residence time inside the knee joints using an in vivo imaging system ‐ Perkin Elmer IVIS® Spectrum. The contralateral legs of mice received an equal concentration of Cy7 free dye (Figure S9). The joints receiving free dye injections had 7% of the initial injected dose remaining on day 3, whereas the Cy7 MPs injected group showed fluorescent signal even on day 35 (Figure S9c). The loss of injected free dye could be attributed to the clearance by the lymphatics in the joints or degradation and loss of fluorescence. The joints did not show any gross signs of inflammation, infections, or difficulty in movements during the entire course of this study. These results align with our previously published work, indicating that dyes encapsulated in 1 μm particles exhibit higher residence time inside mice and rodent knee joints than free dyes.^37^, ^50^ These results suggest that free Cy7 dye (with comparable Mw and LogP to rapamycin) exhibit prolonged joint residence time (~35 days) when encapsulated in MP formulation compared to the free dye.

### 2.7 RMPs prevented the progression of OA disease in a murine post‐traumatic model of OA

**FIGURE 4:**
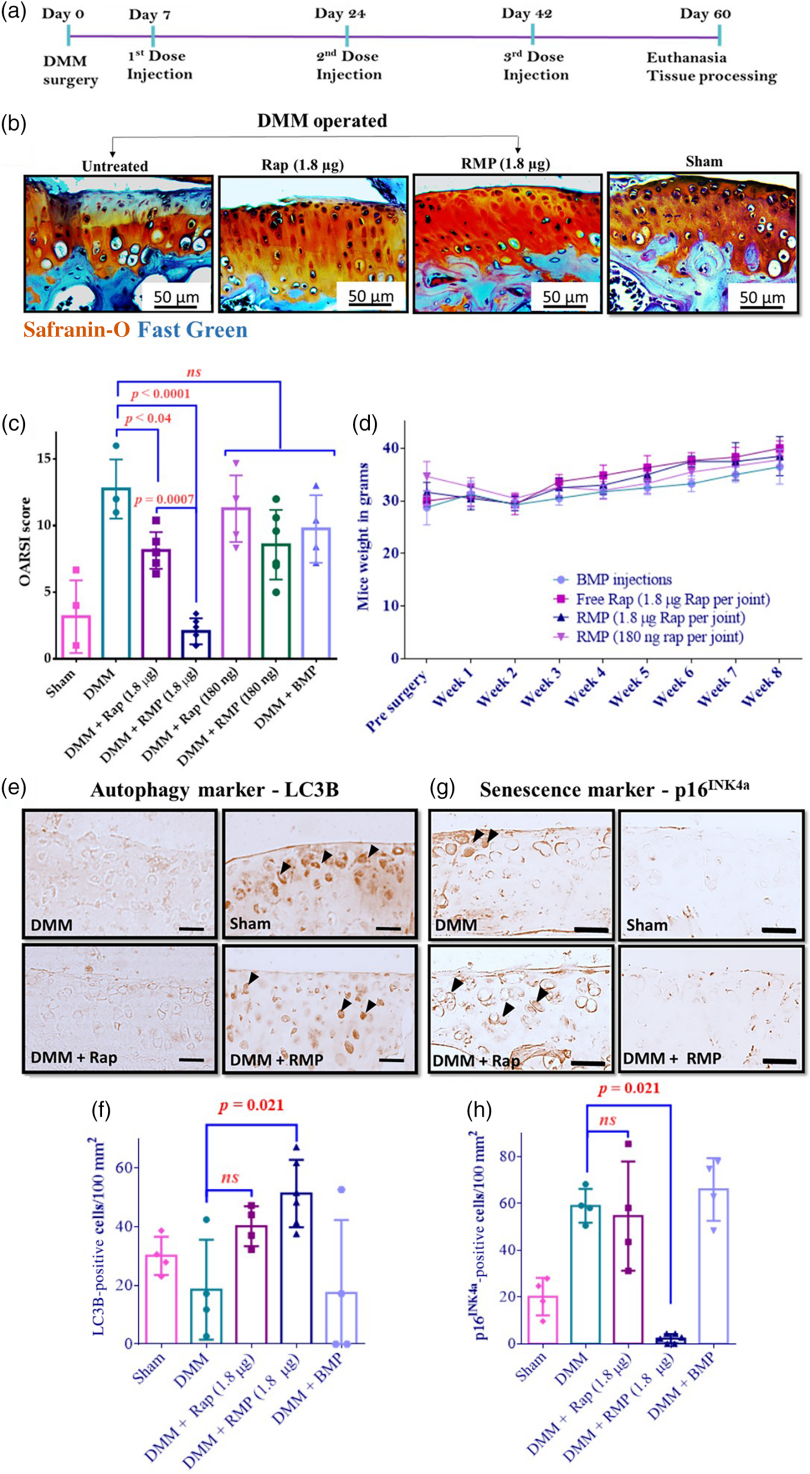
Rapamycin‐loaded microparticles (RMPs) as a prophylactic regimen prevented the progression of osteoarthritis (OA) in the murine post‐traumatic model of OA. (a) Schematic representation of prophylactic treatment timeline. (b) Representative Safranin‐O and Fast Green‐stained images of the medial tibial plateau (MTP) of mice knee joints were collected from different experimental groups. (c) Mouse OARSI scores show the extent of injury in mice after receiving different treatments. (d) Plot showing body weight of mice with respect to time after receiving different treatments. (e) Representative images of LC3B IHC staining. (f) Quantification of LC3B‐positive cells per 100 mm^2^. (g) Representative images of p16^INK4a^ IHC staining. (h) Quantification of p16^INK4a^‐positive cells per 100 mm^2^. The data in the graphs represent the mean ± SD and *p* values were determined using one‐way analysis of variance (ANOVA) or Kruskal–Wallis test and Tukey's post hoc analysis. Destabilization of the medial meniscus (DMM; *n* = 4), sham (*n* = 4), rapamycin (high dose, *n* = 6; low dose *n* = 5), RMPs (*n* = 6), blank microparticles (BMPs; *n* = 4). *ns*, nonsignificant. Scale bar = 50 μm

To test our formulations on a functional model of OA, we utilized a widely used post‐traumatic OA model in mice (destabilization of the medial meniscus [DMM]). In this model, mice are operated to destabilize the medial meniscus by incising the medial meniscotibial ligament and then left free to move in their cages, leading to the development of severe OA by 8 weeks.^58^, ^59^ To test the prophylactic capacity of the formulation, we administered Rapamycin (Rapa), RMP, and blank micro particle (BMP) intra‐articularly in two doses, starting 1 week after the DMM (Figure 4a). To determine an optimal dose of rapamycin, we assessed two different doses (1.8 μg and 180 ng per joint) based on previously published literature.^30^, ^33^ The in vitro release curve of rapamycin from PLGA particles (75–85 kDa PLGA; Figure 2c) showed that 90% of the drug gets released in about 20 days; hence the injection time points (18–20 days apart) were chosen accordingly. The cartilage surface appeared irregular, chondrocytes exhibited focal perichondral staining, and empty lacunae indicating chondrocyte death were observed in the BMPs and free rapamycin‐treated groups. RMPs at the dose of 1.8 μg per joint effectively prevented OA as the cartilage surface appeared smooth, and there was minimal loss of Safranin stain. The maintenance of Safranin‐o staining suggests that the proteoglycan content was maintained, and there were fewer hypertrophic cells than vehicle‐treated groups (Figure 4b,c). The OARSI scores of RMPs (1.8 μg dose) treated mice were fourfold (*p* = 0.0007) and sixfold (*p* ≤ 0.0001) lower than the free rapamycin (1.8 μg dose) treated and DMM mice groups, respectively. The OARSI scores of BMP‐treated mice were not different from DMM‐operated and free rapamycin‐treated groups and were statistically different from RMP‐treated mice OARSI scores (*p* = 0.0003). The lower dose of rapamycin (180 ng per joint), both as a free drug and in MP formulation, failed to prevent the OA disease progression, and hence, for all subsequent studies, we used a dose of 1.8 μg per joint.

Immunohistochemical staining of the knee joints for inflammatory markers (ADAMTS‐5 and MMP‐13), autophagy marker (LC3B), and senescence marker (p16^INK4a^) were carried out on the joint sections. Consistent with our in vitro results, the articular cartilage surface of RMPs (1.8 μg)‐treated mice exhibited higher LC3B‐positive cells indicative of increased autophagy in the cells of the articular cartilage (Figure 4e,f). The free rapamycin‐treated group also exhibited LC3B‐positive cells, although not statistically different from the DMM‐ and BMP‐treated group. The cells positive for p16^INK4a^, a senescence marker, were more than 18‐fold lower in the mice joints treated with 1.8 μg RMPs (Figure 4f,h) than the DMM group (*p* = 0.0212). The reason could be attributed to the sustained and effective inhibition of the mTOR pathway by rapamycin which upregulates autophagy, thus preventing the cells from turning senescent.^30^, ^33^, ^34^, ^38^

Autophagic imbalance in articular chondrocytes is associated with the upregulation of matrix‐degrading enzymes such as matrix metalloproteinases (MMPs) and a disintegrin and metalloprotease with thrombospondin motifs (ADAMTS). Of these proteases, MMP13 and ADAMTS‐5 are critical players in OA progression as they degrade the extracellular matrix and induce hypertrophy of the chondrocytes in the murine knee joints.^60^, ^61^, ^62^, ^63^, ^64^, ^65^ We stained for MMP13 and ADAMTS‐5 using IHC on the articular surface of different treatment groups. In the RMPs (1.8 μg)‐treated groups, the cartilage surface exhibited considerably lower MMP‐13 and ADAMTS‐5‐positive cells than the DMM group (4–6‐fold), and the number of positive cells was comparable to the sham mice group (Figure S10a–j). The DMM, free rapamycin‐treated, and BMP‐treated mice showed much higher MMP‐13 and ADAMTS‐5 staining than RMP‐treated mice.

During the entire treatment regimen, the mice were monitored for drug toxicity‐related weight loss. All animals in the treatment groups showed steady weight gain (Figure 4d).

### 2.8 Therapeutic regimen of RMPs reduces the severity of OA in the murine post‐traumatic model of OA

**FIGURE 5:**
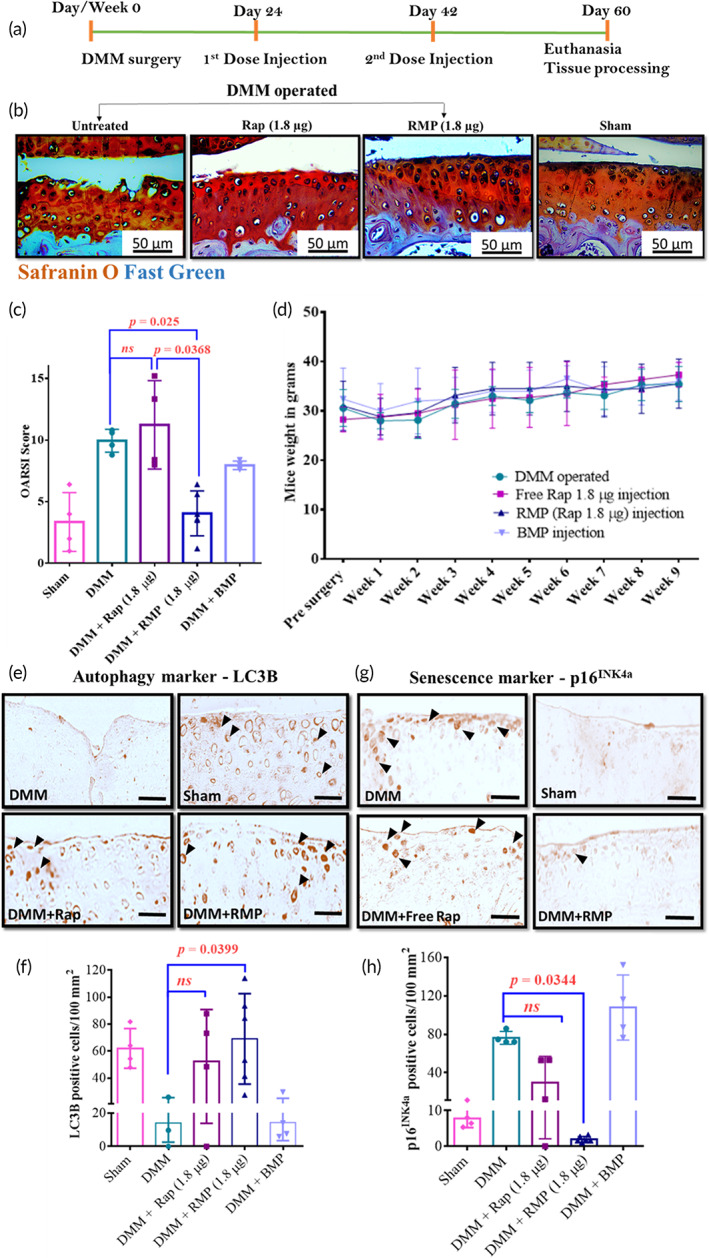
Rapamycin‐loaded microparticles (RMPs) as a therapeutic regimen reduced the severity of osteoarthritis (OA) in the murine post‐traumatic model of OA. (a) Schematic representation of therapeutic treatment timeline. (b) Representative Safranin‐O and Fast Green‐stained images of the medial tibial plateau (MTP) of mice knee joints were collected from different experimental groups. (c) Mouse OARSI scores show the extent of injury in mice after receiving different treatments. (d) Plot showing body weight of mice with respect to time after receiving different treatments. (e) Representative images of LC3B IHC staining. (f) Quantification of LC3B‐positive cells per 100 mm^2^. (g) Representative images of p16^INK4a^ IHC staining. (h) Quantification of p16^INK4a^‐positive cells per 100 mm^2^. The data in the graphs represent the mean ± SD and *p* values were determined using one‐way analysis of variance (ANOVA) or Kruskal–Wallis test and Tukey's post hoc analysis. Destabilization of the medial meniscus (DMM; *n* = 4), sham (*n* = 4), free rapamycin (*n* = 4), RMPs (*n* = 6), blank microparticles (BMPs; *n* = 4). *ns*, nonsignificant. Scale bar = 50 μm

Since most OA patients come to clinics only after experiencing symptoms, we wanted to evaluate whether our rapamycin formulations would be effective once the disease has substantially progressed. It has been shown in the literature that DMM‐operated mice start exhibiting significant early‐stage OA hallmarks, such as proteoglycan loss, chondrocyte hypertrophy, and cartilaginous osteophyte formation, as early as 2 weeks after surgery.^66^ Therefore, we administered the DMM‐operated mice groups with our formulation from 3 weeks post‐DMM surgery and further reduced the number of injections to two (21 days apart) compared to three injections used in our prophylactic study (Figure 5a).

Surgery‐induced OA groups with no treatment, free rapamycin, and BMP injections showed reduced Safranin‐O staining of proteoglycans accompanied by cartilage thinning, surface irregularities, hypertrophic chondrocytes, empty lacunae, and focal perichondral staining (Figure 5b). The OARSI scores of RMP (1.8 μg)‐treated mice were 2.5‐fold lower than the free rapamycin (1.8 μg)‐treated (*p* = 0.0357) and DMM‐only (*p* = 0.0295) mice OARSI scores. RMPs at a dose of 1.8 μg per joint effectively reduced these OA‐associated damages and improved the overall cartilage health.

Consistent with our in vitro results and prophylactic treatment regimen outcomes, the articular cartilage surface of RMP (1.8 μg)‐treated mice exhibited a higher number of cells positive for LC3B, indicating active autophagy in the cells of the articular cartilage. In contrast, the free rapamycin and BMP‐treated groups exhibited fewer LC3B‐positive cells indicative of low basal autophagy (Figure 5e,f). The cells positive for p16^INK4a^ were more than 20‐fold lower in the mice joints treated with 1.8 μg RMPs compared to free rapamycin or DMM‐only (*p* = 0.0344) groups (Figure 5g,h). The free rapamycin‐treated group showed fewer senescent cells than the DMM‐ and BMP‐treated groups but were not statistically different. Consistent with our prophylactic studies, the markers of inflammation, MMP‐13, and ADAMTS‐5‐positive cells on the articular surface of the DMM‐ and BMP‐treated groups were higher in number indicative of cartilage degradation. The cells positive for these catabolic protease markers were more than threefold lower in the RMP‐treated group than free rapamycin‐treated or DMM‐only groups (Figure S11). The results suggest that the sustained presence of rapamycin prevented the cells from secreting these proteases and sustained the ECM production, as evident by lower damage and uniform Safranin‐O staining (Figures 5b,c and S11a–e).

Our studies thus show that the prophylactic and therapeutic regimens using RMPs in our surgically induced OA mice model effectively induced autophagy, prevented senescence, reduced the catabolic proteases in the joints, and paved the way for ameliorating OA in mice models.

## 3 DISCUSSION

Several drugs and biologics like resveratrol, curcumin, torin 1, nordihydroguaiaretic acid, rapamycin, epigenetic modulators, senolytics, and cytokine antagonists have been excellent in modifying OA pathology in animal models but have not entered clinics.^25^, ^31^, ^32^, ^33^, ^42^, ^67^, ^68^ The reason is attributed to the challenges in drug delivery, such as maintaining therapeutic concentrations and preventing rapid clearance of the drug from the targeted area.^5^ Furthermore, in several animal studies, multiple IA injections were used, with some of them administering as high as two IA injections per week which are not clinically translatable. Therefore, the present investigation draws inspiration from two key concepts: the broad literature, which establishes rapamycin as a potent autophagy inducer and senomorphic drug,^30^, ^33^, ^34^, ^37^, ^69^ and engineering a sustained‐release microparticle formulation for clinically acceptable and patient compliant therapy. Here, we were able to show that an IA injection once every 3 weeks was sufficient to treat early OA in mice. This frequency of administration is routinely used in clinics as is the case with Hyalgan® (3 cycles of weekly IA injections for 5 weeks, NCT00669032), Enbrel® (weekly IA injections for 5 weeks, NCT02722772), Traumeel®/Zeel® (weekly IA injections for 3 weeks, NCT01887678), and an ongoing clinical trial with sprifermin (2 or 4 cycles of weekly IA injections for 5 weeks, NCT01919164).

Recent research has shown that micro‐ and nanoparticles can be used to synthesize sustained release systems and can be used to release the disease‐modifying drugs and agents to ameliorate OA.^70^, ^71^ Utilizing such polymer‐based sustained release systems could be the key to clinical translation of disease‐modifying drugs of OA. Since PLGA‐based MPs are clinically used biomaterials with well‐established safety profiles such as Zilretta® that are used for OA pain management, rapamycin is already clinically approved; rapamycin PLGA MPs can be rapidly translated to clinics.

For our animal experiments, we used microparticles synthesized from 75 to 85 kDa molecular weight PLGA which exhibited long residence time (~35 days) in murine knee joints. It is possible that using PLGA particles made from higher molecular weight polymer may exhibit even longer residence time in the joints and further reduce the frequency of administration. Our in vitro rapamycin release profiles found that higher molecular weight PLGA (190–240 kDa) released rapamycin in 120 days compared to about 30 days for 75–85 kDa polymers. Such sustained release platforms should be tested and tuned with appropriate drug loading to continuously release a certain amount of drug and maintain therapeutic concentrations in the joints. Other promising OA drugs can also be codelivered using this platform to explore synergy in OA treatment.

Drugs that exhibit multifaceted approaches such as autophagy activation, senescence prevention, and reducing inflammation in the milieu can potentially turn into promising therapies that are translatable.^72^, ^73^ Autophagy is a stress survival mechanism that gets activated as an adaptive process to different forms of metabolic stress, and the role of autophagy in OA is widely studied.^8^, ^12^, ^15^ Cellular senescence is another growing field and is an important target for many diseases, including OA.^48^, ^74^, ^75^, ^76^ In the mice model of OA, fewer cells were positive for LC3B (Figures 4 and 5e,f), and a higher number of cells were positive for the senescence marker ‐ p16^INK4a^ (Figures 4 and 5g,h) compared to the sham surgery group. In contrast, the RMP‐treated groups upregulated autophagy (Figures 4 and 5e,f) and reduced the expression of p16^INK4a^ (Figures 4 and 5g,h) and chondrocyte hypertrophy markers (MMP‐13 and ADAMTS‐5 expression). Similarly, in vitro, RMP‐induced autophagy, successfully rescued oxidatively stressed primary HACs obtained from OA patients, from turning into senescent phenotypes, and sustained the production of sGAG (Figures 2d–h and 3), which shows its clinical translation potential.

Many studies have highlighted the role of immune cells, such as T cells and B cells, in OA.^77^, ^78^ One of the emerging areas of investigation is evaluating the role of these immune cells, especially T cells, in OA progression. T helper (Th) cells such as Th1, Th9 and, Th17 are known to be present at a significantly higher number in OA joints.^79^ Studies show a positive feedback loop among Th17 cells, IL‐17, and senescent synovial cells, with mTOR playing a significant role in upregulating the production of several cytokines, and clearing the Th17 cells can help in OA disease amelioration.^24^, ^80^ Rapamycin, an immunosuppressive agent and mTOR signaling inhibitor, could directly act on immune cells and potentially reduce the secretion of many inflammatory cytokines such as IL‐17, and thus can prevent protease‐mediated destruction of cartilage.^34^, ^35^, ^80^, ^81^, ^82^ Further experiments are needed to determine whether rapamycin modulates Th cells and IL‐17 production in OA. Since OA is predominantly a geriatric disease, and aged mice report a higher senescence burden and develop more severe OA after injury, additional evaluation into aged mice and genetically modified OA models, such as STR/Ort mice, can broaden the scope of our formulation.^83^, ^84^ Preclinical investigations are also necessary in larger animal models that may require additional optimization of dosages and treatment regimens before translation to humans.

In summary, we report the development of rapamycin as an MP formulation that can be administered after articular joint injury to prevent OA. This formulation was potent to prevent senescence and induced autophagy in articular chondrocytes, reducing inflammatory markers, thus helping prevent and treat post‐traumatic OA in the mice model. The relevance of our findings to human OA was validated using chondrocytes isolated from osteoarthritic patients. These findings provide new insights into senescence prevention and autophagy activation therapies via sustained release formulation to treat trauma‐induced OA.

## 4 MATERIALS AND METHODS

### 4.1 Materials

Poly(D,L‐lactic‐co‐glycolic) acid (PLGA, 50:50) of different molecular weights from 10–15, 75–85, 85–100, and 190–240 kDa (85:15) with carboxylic acid end groups were purchased from Akina (AP041, AP089, AP036) (West Lafayette) and Sigma (739979), respectively. Poly(vinyl alcohol) (PVA, 87–89% hydrolyzed, Mw 13,000–23,000 kDa) was purchased from Sigma (363170). Rapamycin (or Sirolimus, 99.5% purity) was procured from Alfa *Ae*sar (J62473). Insulin/transferrin/selenium purchased from Gibco was used for micromass culture of human chondrocyte cell line (C28/I2). All other chemicals were procured from Sigma Aldrich and Thermo Scientific as analytical grade and used as received. Surgical sutures (poly[glycolic acid]‐based absorbable sutures with ¾ reverse cutting‐edge needle) were obtained from Dolphin sutures.

### 4.2 Antibodies used

Rabbit Anti MMP‐13 polyclonal antibody (Abcam, ab39012), Rabbit Anti ADAMTS‐5 polyclonal antibody (Abcam, ab41037), Rabbit Anti p16^INK4a^ polyclonal antibody (Thermo Fischer, PA1‐30670), Rabbit Anti LC3B polyclonal antibody (Thermo Fischer, PA1‐16930), Alexa Fluor 488 goat anti‐rabbit IgG (H+L) (Abcam, A11008), and HRP conjugated goat anti‐rabbit secondary antibody (Thermo Fischer, 31460).

### 4.3 Cell line and culture conditions

The human immortal chondrocyte cell line – C28/I2 (Merck) was used in this study. Cells were cultured in DMEM/F‐12 (Ham) media (Invitrogen) containing 1 mM sodium pyruvate, 10 mM of HEPES, 140 mM of glucose. The media was supplemented with 10% fetal bovine serum (US origin, Sigma) and 1% antibiotic cocktail containing penicillin/streptomycin (Invitrogen) in an atmosphere of 5% CO_2_ and 37°C.

### 4.4 Mice

All the animal experiments were approved by the Institutional Animal Ethics committee (IAEC) (CAF/Ethics/612/2018 and CAF/Ethics/808/2020). IAEC guidelines were followed for the design, experimentation, and analysis of animal experiments. Mice were housed at the Central Animal Facility, Indian Institute of Science (IISc) in individually ventilated cages under monitored temperature and humidity with automated 12 h light–dark cycles. Mice were fed with standard laboratory food and water. Female wild‐type (WT) mice of C57BL/6 strain were used for IA injection studies, and male WT mice of C57BL/6 strain were used for OA studies. Post‐traumatic OA induced via DMM surgery causes joint damage in both female and male mice, although it is reported that it is less severe in females,^85^, ^86^ and hence we used male mice for all surgery‐based OA studies.

### 4.5 Human cartilage samples

All the human ex vivo experiments were approved by the Institutional Human Ethics committee (IHEC) (02/31.03.2020) and MS Ramaiah Medical College, Ethics Committee (MSRMC/EC/AP‐06/01‐2021). IHEC and MSRMC/EC guidelines were followed to design, experiment, and analysis of human experiments. Human knee joint surfaces from the femur and tibia were obtained from patients undergoing total knee replacement due to end‐stage OA from Ramaiah Medical College and Hospital.

### 4.6 Sample's collection consent statement

The human articular cartilage samples collected to perform this study were obtained from patients with OA undergoing total knee arthroplasty from MS Ramaiah Medical College according to IISc‐ and MSRMC‐approved ethical protocol. Patients in the registry at MSRMC gave informed consent to donate knee joints for research. The information about the study and informed consent sheet was provided to the participants in the research. The information obtained by the analysis of the samples was always treated under confidentiality. The details about the volunteers used in the study are listed in Table S2.

## 5 METHODS

### 5.1 Synthesis of PLGA microparticles

PLGA microparticles were synthesized from PLGA polymer of various molecular weights, using the single emulsion technique described earlier.^37^ Briefly, 100 mg of PLGA was dissolved in 2 mL of dichloromethane (DCM) with or without rapamycin (1 mg), and the homogenization was carried out in 1% polyvinyl alcohol (10 mL) at 12,000 rpm. This solution was added to 1% PVA (110 mL) and was allowed to stir continuously for 3–4 h to evaporate DCM completely. The solution was then centrifuged at 11,000 × *g*, and the pellet was washed using deionized water twice to wash away the excess PVA. The microparticles were then resuspended in deionized water and rapidly frozen at −80°C, followed by lyophilization. The lyophilized particles were used for in vitro and in vivo studies by making a suspension in 1x PBS according to required concentrations and sterilizing them under UV for 20 min. For determining the size of PLGA microparticles, 50 μg particles were dispersed in deionized water (1 mL) and sonicated briefly before analysis. Particle size distributions were determined using dynamic laser light scattering (Nano ZS ZetaSizer; Malvern Instruments).

## 6 PHYSIOCHEMICAL CHARACTERIZATION OF THE MICROPARTICLE FORMULATION

### 6.1 Encapsulation efficiency

To estimate the amount of rapamycin encapsulated in PLGA particles, a standard curve was first prepared by adding different concentrations of rapamycin in DMSO solution containing PLGA (10 mg mL^−1^). The absorbance was read using an SPL UV Max™ plate (33096) at 278 nm using a plate reader (Tecan™ Microplate Spectrophotometer). The rapamycin‐PLGA microparticles (10 mg mL^−1^) were weighed and dissolved in DMSO, and the amount of rapamycin encapsulated was determined from the standard curve. EE was calculated using the following equation:

$$\text{Encapsulation Efficiency}=\frac{\text{Amount of Rapamycin encapsulated}}{\text{Total amount of Rapamycin added}}\times 100$$

### 6.2 In vitro release profile of rapamycin/Cy7‐loaded microparticles

Rapamycin or Cy7‐loaded microparticles of different molecular weights (1 mg) were suspended in 1x PBS (1 mL) solution (pH 7.4) at 37°C in a rotatory spinner. The particles were pelleted at 11,000 ×*g* and dissolved in 100 μL DMSO at different incubation times. The dissolved particles were quantified through a plate reader instrument (Tecan™ Microplate Spectrophotometer) with predetermined parameters set to record the absorbance at 278 nm for rapamycin and fluorescence intensity at 750/773 nm for Cy7 dye. The amount of rapamycin or Cy7 released at each time point was determined from the standard curve made using PLGA spiked with rapamycin or Cy7. The experiment was carried out in triplicates along with blank microparticles as controls. The PLGA particles were loaded with Cy3 dye (50 μg) for in vitro imaging.

### 6.3 Isolation of HACs from human knee joint surfaces

Knee joints excised during surgery were immediately placed in sterile containers with 1x PBS and brought to the tissue culture facility within an hour. The human articular knee joint surfaces were briefly rinsed in 1x PBS (containing 1% penicillin–streptomycin) once before scraping the cartilage from the underlying bone surface into thin slices of 1–3 mm^3^. These slices were placed in a 10 cm dish and were washed three times with 1x PBS. The slices were then incubated at 37°C for 30 min in DMEM‐F12 media containing pronase (1 mg mL^−1^). Slices were again rinsed with 1x PBS and were incubated at 37°C for 16–18 h in DMEM‐F12 media containing collagenase A solution (3 mg mL^−1^). After 16–18 h, the clumps were broken apart using a pipette, and the cells were pelleted down at 1000 × *g* for 10 min. The cells were washed with 1x PBS thrice. Finally, the cells were resuspended in DMEM‐F12 media, counted, and seeded at a density of 1 × 10^6^ cells mL^−1^ in T75 cm^2^ (13333.33 cells cm^−2^) cell culture flasks. The media was changed every 2 days, and the cells were subsequently subcultured and used for experiments within the first two passages. All the chondrocyte experiments were performed using transwell inserts to prevent direct contact and uptake of microparticles.

### 6.4 Senescence induction assay

Primary HACs were plated in a 24‐well plate with 15,000 cells per well in the untreated group and 30,000 cells per well for other treatment groups. The chondrocytes (C28/I2) were plated in a 24‐well plate with 7500 cells per well in the untreated group and 15,000 cells per well for other treatment groups to maintain cells under subconfluency until the end of the experiment. The difference in the number of cells being plated for senescence assay ensures that the cell numbers are equivalent in all groups at the end of the experiment. We seeded fewer cells in untreated wells as the senescent cells do not proliferate while the untreated cells proliferate to become confluent. Since confluent cells exhibit contact inhibition and stain positive for SA β Gal, the lower plating density ensured that cells remained subconfluent at the end of the 48‐h experiment.^87^ They were treated with different stress conditions, such as genotoxic stress agent BrdU (200 μM) or oxidative stress agent hydrogen peroxide (H_2_O_2_) (200 μM) to simulate inflammation in post‐traumatic OA.

The primary treatment groups were vehicle‐treated cells (DMSO), BrdU‐treated cells or H_2_O_2_‐treated cells, BrdU‐treated cells, or H_2_O_2_‐treated cells along with free rapamycin (1 μM) cotreated cells, BrdU‐treated cells or H_2_O_2_‐treated cells along with RMPs (equivalent to 1 μM) cotreated cells, BrdU‐treated cells or H_2_O_2_‐treated cells along with BMP cotreated cells. Wherever microparticles were added to the treatment groups, transwell inserts were used to prevent direct contact of microparticles with chondrocytes. Colorimetric SA‐β Gal activity was used to stain for senescent cells as previously described.^88^ After exposing the cells to these stress agents for 48 h, cells were washed thrice with 1x PBS and then fixed with a fixative solution containing 2% formaldehyde and 0.2% glutaraldehyde in 1x PBS for 10 min. Following fixation, cells were incubated in SA‐β Gal staining solution (1 mg mL^−1^ 5‐bromo‐4‐chloro‐3‐indolyl‐beta‐d‐galactopyranoside [X‐Gal], 1x citric acid/sodium phosphate buffer [pH 6.0], 5 mM potassium ferricyanide, 5 mM potassium ferrocyanide, 150 mM sodium chloride, and 2 mM magnesium chloride) at 37°C overnight. The enzymatic reaction was stopped after 16 h, and cells were washed three times with 1x PBS. Five random brightfield images were taken per well. These images were analyzed using ImageJ software.

To automatically score the senescent cells, we developed a custom‐built macros algorithm to score the senescent cells based on their size and the intensity of SA‐β Gal staining. Bright‐field images were taken from each treatment group (vehicle—1x PBS for H_2_O_2_‐treated group and DMSO for BrdU‐treated group). The macros algorithm was run to count the total number of senescent cells.

### 6.5 RMPs treatment in senescence induction assays (micromass culture)

We generated micromasses as described previously.^55^ Briefly, the cells were seeded as a 15 μL suspension in growth media in a 24‐well plate at a density of 2.5 × 10^7^ cells mL^−1^. The cells were allowed to adhere to the well plate for 3 h, after which growth media was added. After 24 h, the growth media was changed to differentiation media containing supplements (insulin/transferrin/selenium, TGF‐β [10 ng mL^−1^], and ascorbic acid). Micromasses were treated with BrdU (600 μM) or H_2_O_2_ (100 μM) in individual experiments and cotreated with free rapamycin (1 μM) or RMPs (final concentration 1 μM). After 48 h of incubation, the micromasses were fixed using 4% formaldehyde followed by Alcian blue staining at pH < 1 to stain the sGAG overnight. After 16–18 h, micromasses were washed with deionized water to remove any nonspecific stains, followed by Alcian blue stain extraction using 6M Guanidine HCl. The extracted Guanidine HCl's absorbance was read at 630 nm using a plate reader (Tecan™ Microplate Spectrophotometer) to quantify the sGAG present in the micromasses after various treatments. Similar experiments were carried out to evaluate the long‐term sGAG production with an incubation time of up to 8 days. Media was changed every 2 days and was replenished with BrdU or H_2_O_2_ along with respective treatments.

### 6.6 Immunocytochemistry

After 48 h of treatment, the cells were fixed using 4% PFA followed by permeabilization using 0.05% Triton X‐100. The cells were then blocked using 5% nonfat dry milk and incubated overnight at 4°C with rabbit anti‐LC3B polyclonal antibody (0.5 μg mL^−1^). After 16–18 h, the cells were washed and incubated with Alexa Fluor 488 goat anti‐rabbit IgG (H + L) (1 μg mL^−1^) for 1 h followed by nuclear staining using DAPI (300 nM) for 10 min. These cells were then visualized using FITC (495/519 nm) and DAPI (358/451 nm) channel using IN Cell Analyzer 6000 (GE Life Sciences) at 40× magnification.

The obtained images were quantified for the LC3B puncta per cell using a custom‐built automated macros algorithm. For counting LC3B puncta, the FITC channel images threshold was adjusted to pick up and count the individual puncta. The same threshold values were used for the analysis of all the images in the experiment. Similarly, the total cell numbers were obtained by an automated macro algorithm picking up the DAPI stained nucleus to give the total cell number. The average puncta per cell were obtained by dividing the total puncta per image by the total number of cells in that image.

### 6.7 Residence time of PLGA MPs in mouse knee joint

PLGA microparticles of molecular weight 75–85 kDa with Cy7 dye were suspended in 1x PBS at a concentration of 20 mg mL^−1^. Ten microliters of this formulation was injected into mice's left knee joint by IA injections on day 0. The contralateral legs were used as control and were given an equivalent dose of free Cy7 dye. The mice (*n* = 5 per group) were anesthetized by isoflurane prior to transfer in the imaging system (IVIS® Spectrum In Vivo Imaging System), and the fluorescence signal was measured with predetermined exposure time. Imaging (fluorescence) was performed on day 0 (pre‐ and post‐injection), 1, 3, 5, 7, 14, 21, 30, and 35 using the IVIS. Radiance efficiency (p s^−1^ sr^−1^ μW^−1^) within a region of interest was quantified by the IVIS® Spectrum Living imaging software.

### 6.8 Effect of RMPs on surgically induced OA

Male WT C57BL/6 mice (25 g) were used for this study.^86^ Following intraperitoneal injection of ketamine (80 mg kg^−1^) and xylazine (10 mg kg^−1^) as a combination in sterile 1x PBS, the animals were rested until the surgical plane was reached with no active plantar reflexes. DMM was performed in mice knee joints to induce post‐traumatic OA. A medial parapatellar incision exposed the knee joint capsule. The patella was displaced laterally after the joint capsule was opened, followed by transection of the medial meniscotibial ligament using surgical blade number 11 and scalpel. Following irrigation of the operated site with saline, the capsule and skin were sutured separately using absorbable PGA sutures. Sham groups received only an incision to expose the joint capsule, then sutured back like the other DMM‐operated groups. The mice were not immobilized and could move freely in the cage postoperatively. Each experimental group was evaluated by gross morphological examination for swelling, pain, or change in gait of the animal postsurgery and during the entire duration of the experiment.

### 6.9 Classification of study

Prophylactic dose study:

Group 1: DMM operated animals with no treatment (4 mice).

Group 2: Surgical control receiving no treatment (Sham) (4 mice).

Group 3: DMM operated with free rapamycin injection (1.8 μg) (6 mice).

Group 4: DMM operated with free rapamycin injection (180 ng) (5 mice).

Group 5: DMM operated with RMP injection (200 μg particles containing the equivalent of 1.8 μg rapamycin) (6 mice).

Group 6: DMM operated with RMP injection (200 μg particles containing the equivalent of 180 ng rapamycin) (6 mice).

Group 7: DMM operated with BMP injection (200 μg particles) (4 mice).

Therapeutic dose study:

Group 1: DMM operated animals with no treatment (4 mice).

Group 2: Surgical control receiving no treatment (Sham) (4 mice).

Group 3: DMM operated with free rapamycin injection (1.8 μg) (4 mice).

Group 4: DMM operated with RMP injection (200 μg particles containing the equivalent of 1.8 μg rapamycin) (6 mice).

Group 5: DMM operated with BMP injection (200 μg particles) (4 mice).

In the prophylactic study, IA injection was given 7 days after DMM surgery, followed by injections at days 24 and 42 with euthanasia at day 60. In the therapeutic study, the first injection was given on day 24 after DMM surgery, followed by a second injection on day 42 with euthanasia at day 60.

### 6.10 Histology

The joints of operated knees were fixed in 4% paraformaldehyde for 24 h. The joints were embedded in paraffin after decalcification in 5% formic acid for 5 days. Five‐micron sections were stained with Safranin‐O‐fast green staining using the described protocol.^89^ In brief, slides were deparaffinized with xylene and alcohol. After a 5‐min wash in running tap water, slides were stained with fresh Wiegert's iron hematoxylin for 10 min. Following a 5‐min wash in running tap water, slides were soaked in 0.1% Safranin‐O (10 min) and 0.05% fast green (5 min) in this order. After dehydration with alcohol and xylene, the slides were mounted with coverslips using mounting media. The medial tibial plateaus were imaged in the prepared slides and graded according to the OARSI OA cartilage histopathology assessment system.^90^ Veterinarians performed the scoring in a blinded manner to minimize observer bias.

Similar steps were followed for hematoxylin and eosin staining as described above until Wiegert's iron hematoxylin stain. Following a 5‐min wash in running tap water, slides were soaked in 1% eosin staining solution for 5 min followed by rinsing under tap water for 5 min. After dehydration with alcohol and xylene, the slides were coverslipped using mounting media, and the synovial lining of the different treatment groups was imaged.

### 6.11 Immunohistochemical studies

Sections were processed similar to Safranin‐O staining until the alcohol step followed by heat‐induced epitope retrieval at 95°C in 1x TBST buffer (pH 9), followed by blocking using nonfat dry milk (0.8%). The slides were rinsed in 1x TBST and incubated with MMP‐13, ADAMTS‐5, LC3B, and p16^INK4a^ antibodies (1:300) diluted in blocking solution at 4°C for 16–18 h. The slides were again washed in 1x TBST followed by incubation with HRP conjugated secondary antibody (1:150) diluted in blocking solution at room temperature for 2 h. The slides were again rinsed, and DAB (3,3′‐diaminobenzidine) substrate was added and incubated for 1 h. Before brightfield imaging, the slides were washed, dried, and coverslipped using mounting media. The number of cells positive in each image was quantified using an automated macros program in ImageJ software.

### 6.12 Statistical analysis

All statistical analysis was performed using Graph Pad Prism 6. Data were presented as mean ± SD. Nonlinear regression (least square method) two‐phase exponential decay curve was used to fit rapamycin release profile with constrains of maximum value as 100 and minimum value as 0. Differences between groups were analyzed by *t* test or one‐way analysis of variance (ANOVA), and nonparametric data were analyzed using the Mann–Whitney *U* test or Kruskal–Wallis test with Tukey's/Dunn's multiple comparison test, with *p* < 0.05 considered significant. Since the mean and SD estimate for each experimental animal group was not available initially, we kept a minimum of four animals in each group. After the results were available, we retrospectively calculated the statistical power to detect differences between free rapamycin and RMPs treatment groups using G*Power 3.1 software. The power for both prophylactic and therapeutic mice OA studies was greater than or equal to 80%.

### 6.13 Data availability

All data generated or analyzed during this study are included in this published article (and its Supplementary Information files).

### 6.14 Code Availability

The macros code used for analysis during the current study are available from the corresponding author on reasonable request.

## AUTHOR CONTRIBUTIONS

**Kaamini Dhanabalan:** Conceptualization (supporting); data curation (lead); formal analysis (lead); investigation (lead); methodology (lead); project administration (supporting); resources (supporting); software (lead); validation (lead); visualization (lead); writing – review and editing (supporting). **Ameya A. Dravid:** Methodology (supporting); writing – review and editing (equal). **Smriti Agarwal:** Investigation (supporting); methodology (supporting); writing – review and editing (supporting). **Ramanath K. Sharath:** Resources (supporting); writing – review and editing (equal). **Ashok Kumar Padmanabhan:** Resources (supporting); writing – review and editing (supporting). **Rachit Agarwal:** Conceptualization (lead); investigation (supporting); project administration (lead); resources (lead); software (supporting); supervision (lead); validation (supporting); visualization (supporting); writing – review and editing (lead).

## CONFLICT OF INTERESTS

The other authors declare that they have no competing interests.

### PEER REVIEW

The peer review history for this article is available at https://publons.com/publon/10.1002/btm2.10298.

## Supporting information


**Appendix S1:** Supplementary Information

## Data Availability

The data that support the findings of this study are available from the corresponding author upon reasonable request.
