# Supplementary material for: Intra‐articular injection of rapamycin microparticles prevent senescence and effectively treat osteoarthritis
Source: Bioeng Transl Med. 2022 May 5;8(1):e10298. doi: 10.1002/btm2.10298 (PMC9842044; doi:10.1002/btm2.10298)
Supplement: Supplementary file 1 — Appendix S1: Supplementary Information [file BTM2-8-e10298-s001.docx]

**Intra-articular Injection of Rapamycin Microparticles Prevent Senescence and Effectively Treat Osteoarthritis**

Kaamini M. Dhanabalan^1^, Ameya A. Dravid^1^, Smriti Agarwal^1^, Ramanath K Sharath^2^, Ashok Padmanabhan^2^, Rachit Agarwal^1*^

*Author’s affiliations:*

*^1^Centre for BioSystems Science and Engineering, Indian Institute of Science, Bengaluru, India 560012.*

*^2^Department of Orthopedics, MS Ramaiah Medical College, Bengaluru, India 560054.*

Corresponding author’s email: rachit@iisc.ac.in

**Supplementary information**

Figure S1. Representative human tibial articular surface bearing lesions from OA patients.

Figure S2. Rapamycin MPs induce autophagy in chondrocytes.

Figure S3. Dose optimization to determine the concentration of oxidative stressor (H_2_O_2_) to induce senescence in chondrocytes.

Figure S4. Rapamycin MPs prevent senescence in chondrocytes (C28/I2) under genotoxic stress.

Figure S5. Rapamycin MPs prevent senescence in chondrocytes (C28/I2) under oxidative stress.

Figure S6. Dose optimization to determine the concentration of stress agents to reduce sGAG production in micromasses.

Figure S7. Rapamycin MPs prevent loss of sGAG in micromass cultures exposed to genotoxic and oxidative stresses.

Figure S8. *In vitro* Cy7 release curve follows a similar trend of rapamycin release from PLGA MPs.

Figure S9. PLGA MPs exhibit prolonged residence time in mice knee joints.

Figure S10. RMPs in the prophylactic regimen reduced the markers of inflammation associated with OA in surgically induced murine OA models.

Figure S11. RMPs in the therapeutic regimen reduced the markers of inflammation associated with OA in surgically induced murine OA models.

Table S1: PLGA microparticles of different molecular weights and PLA: PGA ratios, their respective sizes, and rapamycin encapsulation efficiency.

Table S2: Details of volunteers who donated the knee joint samples following total knee arthroplasty.


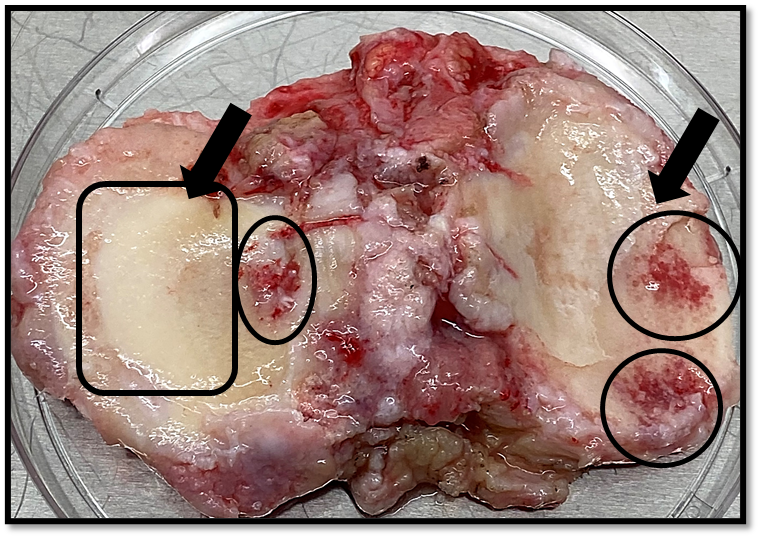


**Figure S1. Representative human tibial articular surface bearing lesions from OA patients.** The circles represent the areas bearing OA lesions with involvement of subchondral bone and the rectangular area represents the non-lesioned areas which were used for IHC.


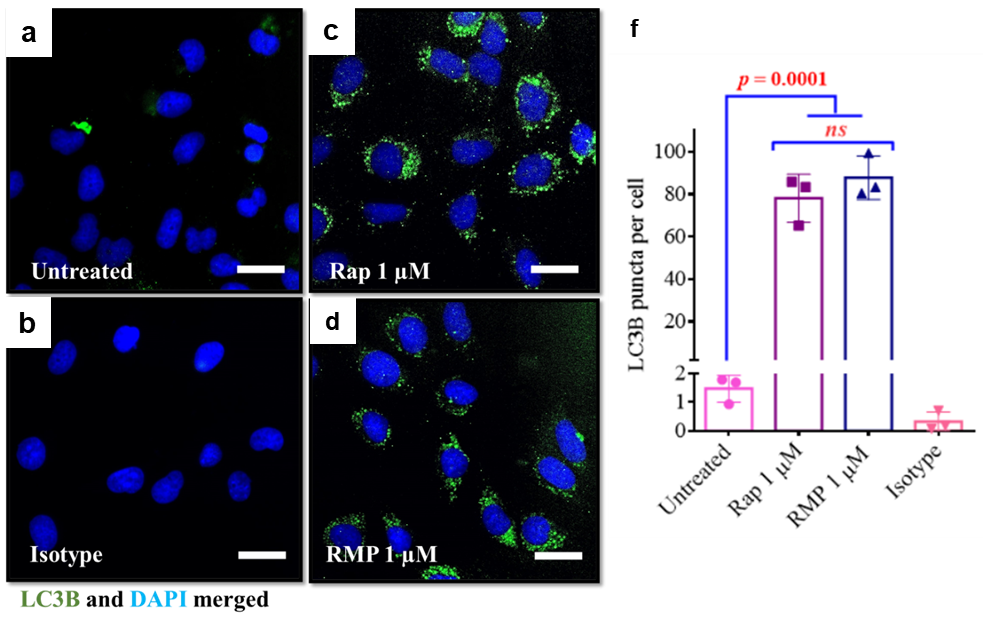


**Figure S2.** **Rapamycin MPs induce autophagy in chondrocytes.** Fluorescence microscopy images of C28/I2 cells stained with DAPI and LC3B after (**a**) no treatment, **(b)** isotype antibody, **(c)** free rapamycin (1 µM) and **(d)** Rapamycin MPs (1 µM rapamycin) treatment. **(e)** Quantification of LC3B puncta per cell using ImageJ software (n = 3 per group). Data in graphs represent the mean ± s.d. and *p* values were determined by one-way analysis of variance (ANOVA) and Tukey’s post hoc tests. *p*-value < 0.05 was considered significant. *ns* - non significant; Scale bar, 20 µm.

**
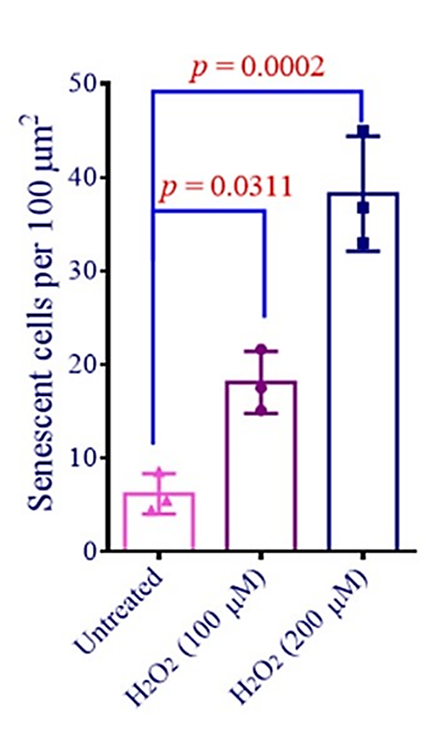
**

**Figure S3. Dose optimization to determine the concentration of oxidative stressor (H_2_O_2_) to induce senescence in chondrocytes.** Graph shows senescent cells per 100 µm^2^ in oxidatively stressed chondrocytes. Data in graph represent the mean ± s.d. and *p* values were determined by one-way analysis of variance (ANOVA) and Tukey’s post hoc. *p*-value < 0.05 was considered significant. *ns* - non significant.


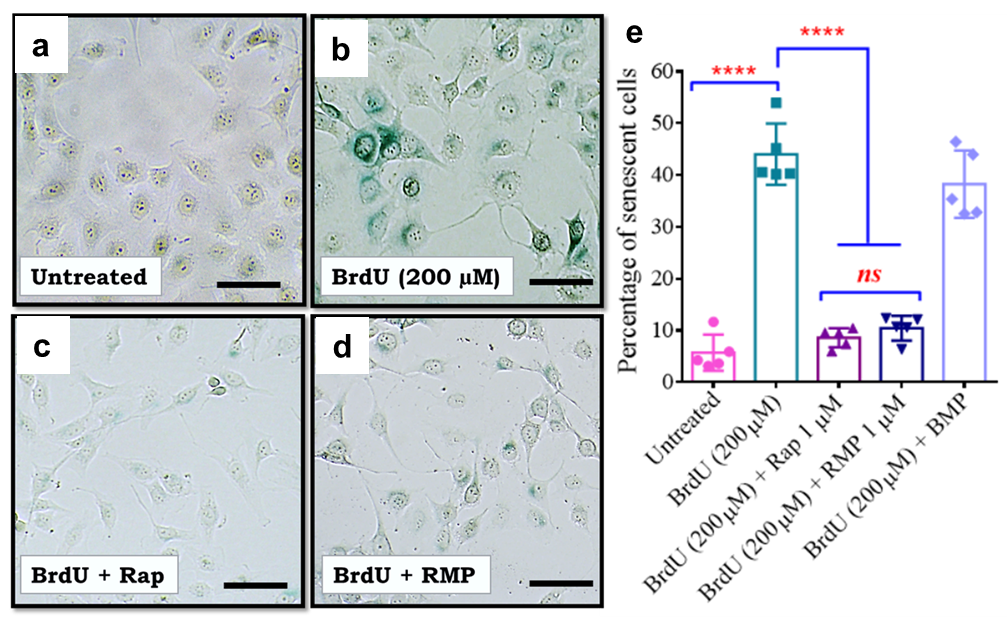


**Figure S4. Rapamycin MPs prevent senescence in chondrocytes (C28/I2) under genotoxic stress.** SA-β Gal-stained images of C28/I2 cells exposed to **(a)** no treatment, **(b)** genotoxic (BrdU) stress, **(c)** genotoxic (BrdU) stress along with free rapamycin (1µM) treatment and, **(d)** genotoxic (BrdU) stress along with rapamycin MPs (1µM) treatment. **(e)** Percentage of senescent cells under genotoxic (BrdU) stress condition, (n = 5 per group). Data in the graph represent the mean ± s.d. and *p* values were determined by one-way analysis of variance (ANOVA) and Tukey’s post hoc tests. *p*-value < 0.05 was considered significant. BMP-Blank Microparticles, RMP- Rapamycin loaded Microparticles. *****p*<0.0001, *ns* - non significant; Scale bar, 40 µm.

**
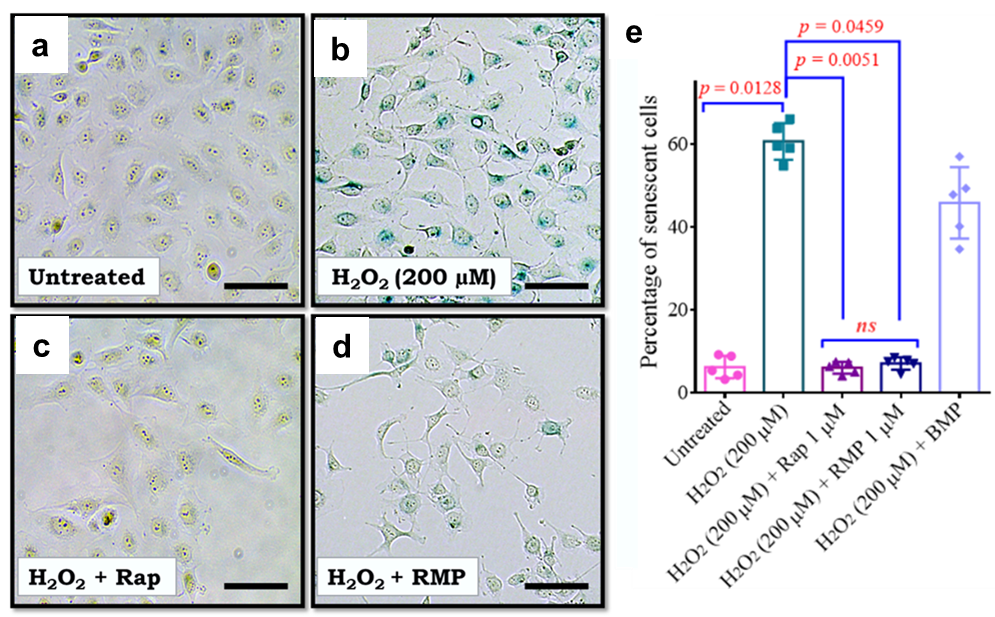
Figure S5. Rapamycin MPs prevent senescence in chondrocytes (C28/I2) under oxidative stress.** SA-β Gal-stained images of C28/I2 cells exposed to **(a)** no treatment, **(b)** oxidative (H_2_O_2_) stress, **(c)** oxidative (H_2_O_2_) stress along with free rapamycin (1µM) treatment and, **(d)** oxidative (H_2_O_2_) stress along with rapamycin MPs (1µM) treatment. **(e)** Percentage of senescent cells under oxidative (H_2_O_2_) stress condition (n = 5 per group). Data in the graph represent the mean ± s.d. and *p* values were determined by one-way analysis of variance (ANOVA) and Tukey’s post hoc tests. *p*-value < 0.05 was considered significant. BMP-Blank Microparticles, RMP- Rapamycin loaded Microparticles. *ns* - non significant; Scale bar, 40 µm.


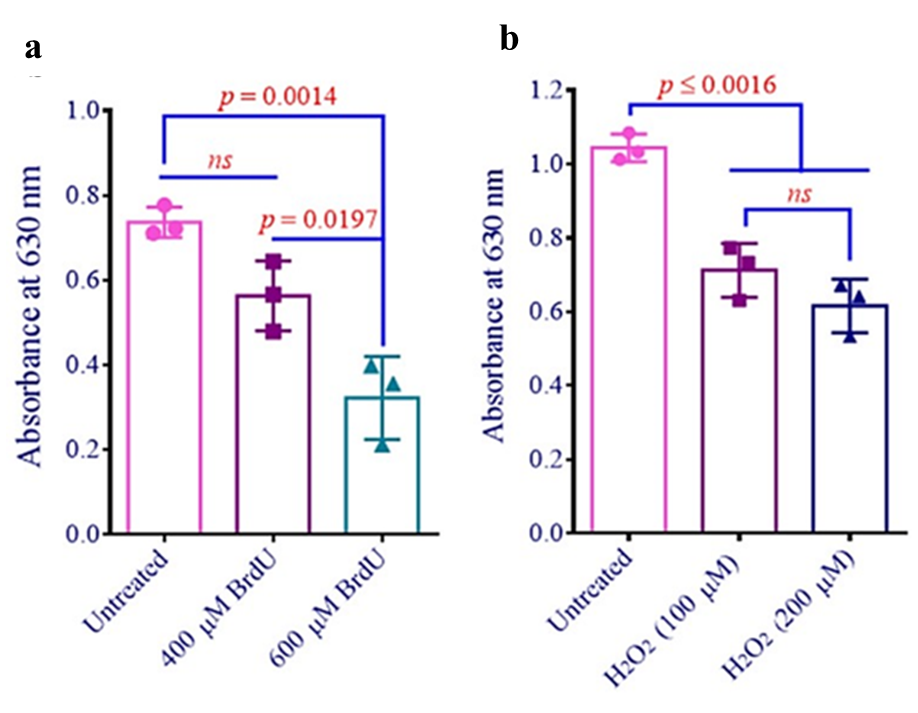


**Figure S6. Dose optimization to determine the concentration of stress agents to reduce sGAG production in micromasses.** sGAG production from C28/I2 micromass culture after treatment with **(a)** genotoxic (BrdU) stress for 48 h (n = 3), **(b)** oxidative (H_2_O_2_) stress for 48 h (n = 3). Data in graphs represent the mean ± s.d. and *p* values were determined by one-way analysis of variance (ANOVA) and Tukey’s post hoc. *p*-value < 0.05 was considered significant. *ns* - non significant.

**
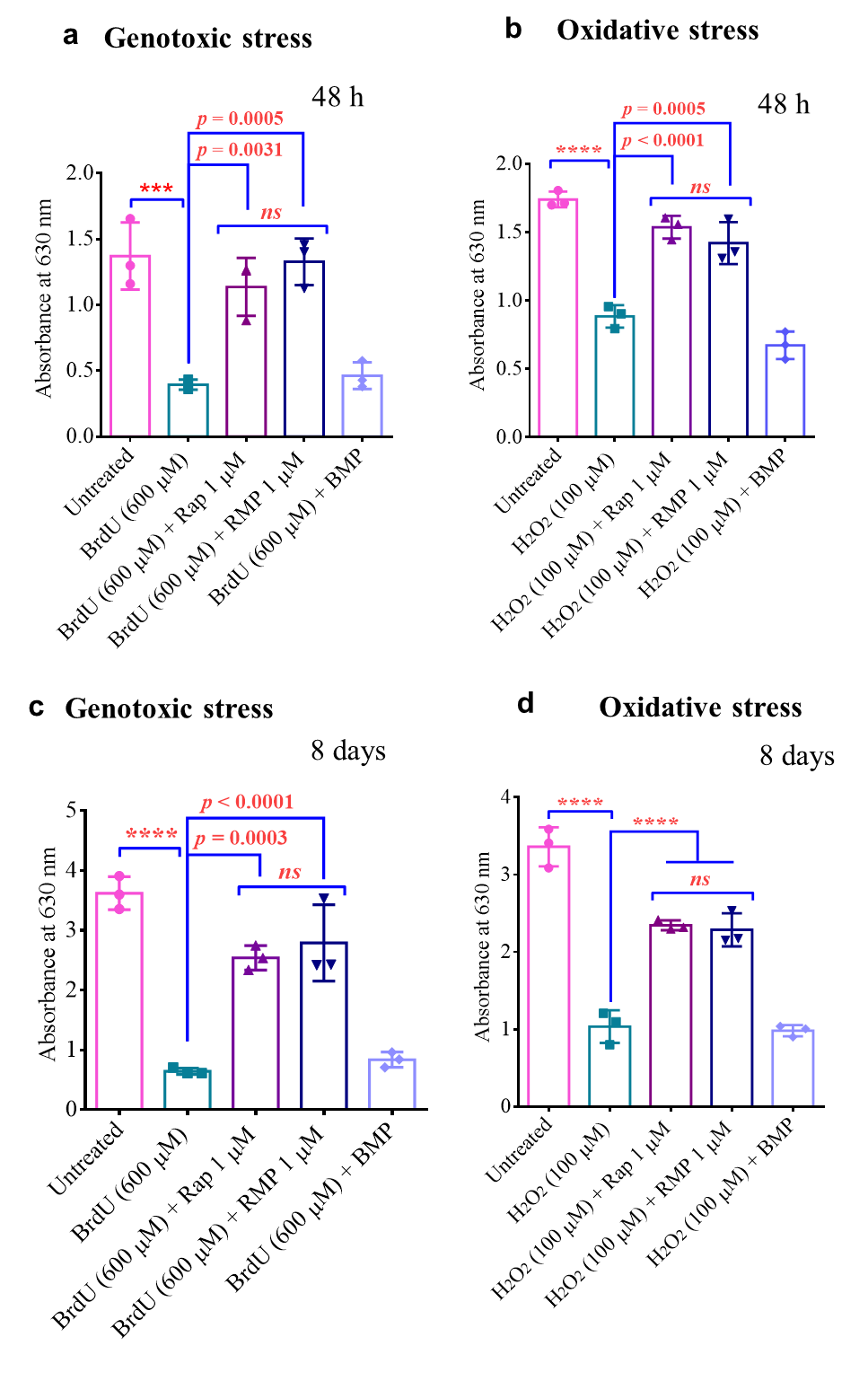
**

**Figure S7.** **Rapamycin MPs prevent loss of sGAG in micromass cultures exposed to genotoxic and oxidative stresses.** sGAG production from C28/I2 micromass culture after treatment with various particle and drug formulations under **(a)** genotoxic (BrdU) stress for 48 h (n = 3), **(b)** oxidative (H_2_O_2_) stress for 48 h (n = 3), **(c)** genotoxic (BrdU) stress for 8 days (n = 3), and **(d)** oxidative (H_2_O_2_) stress for 8 days (n = 3). Data in graphs represent the mean ± s.d. and *p* values were determined by one-way analysis of variance (ANOVA) and Tukey’s post hoc. *p*-value < 0.05 was considered significant. BMP - Blank Microparticles, RMP - Rapamycin loaded Microparticles. *****p*<0.0001, ****p*<0.001, *ns* - non significant.


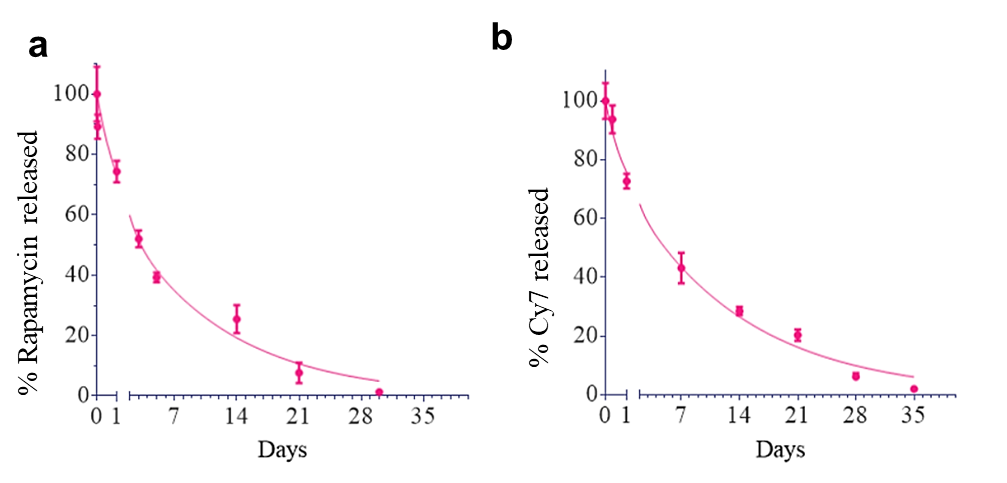


**Figure S8.** **In vitro Cy7 and rapamycin release curve follows a similar trend from PLGA MPs.** Quantification of in vitro release profiles of **(a)** rapamycin and **(b)** Cy7 dye from PLGA particles synthesized from PLGA 75-85 kDa (50:50). Non-linear regression (least square method) was used to fit a two-phase exponential decay curve in all the release profiles. Data in graphs represent the mean ± s.d. (n = 3).

**
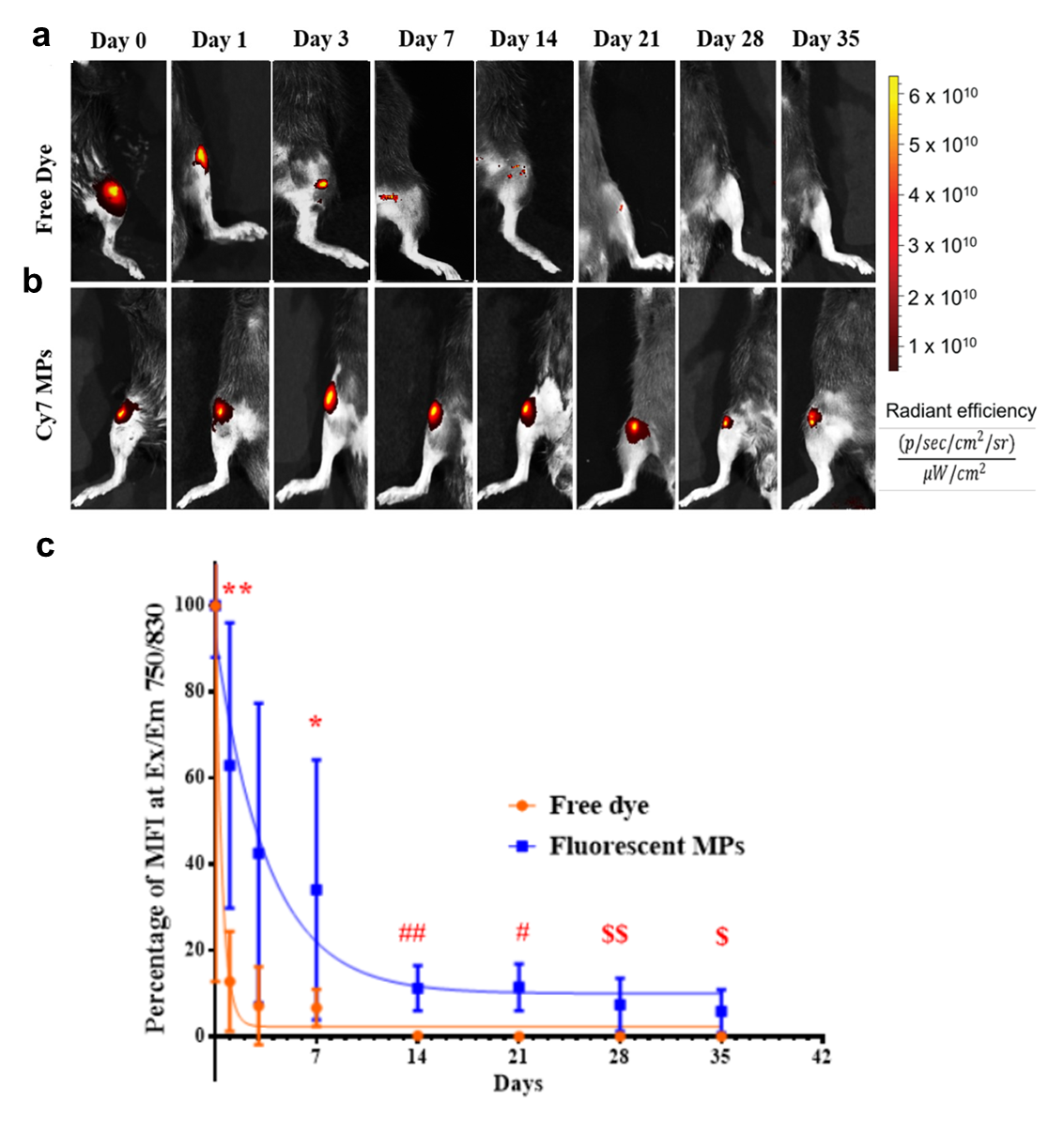
**

**Figure S9. PLGA MPs exhibit prolonged residence time in mice knee joints.** *In vivo* images of mice knee joints imaged at0, 1, 3, 7, 14, 21, 28, and 35 days after intra-articular injection of **(a)** free Cy7 dye and **(b)** Cy7-labeled PLGA MPs. **(c)** Percentage fluorescent intensity remaining of injected formulations with respect to days (n = 5 mice per group). Data in graph were fitted with non-linear regression (least square method) one-phase exponential decay curve. Data in graph represent the mean ± s.d. (n = 5 at each time point) and *p* values were determined by unpaired t test or Mann Whitney test. *p*-value < 0.05 was considered significant. ***** - 0.0159, ******- 0.0126, **#** - 0.0016, **##** - 0.0079, **$** - 0.0296, **$$** - 0.0269.

**
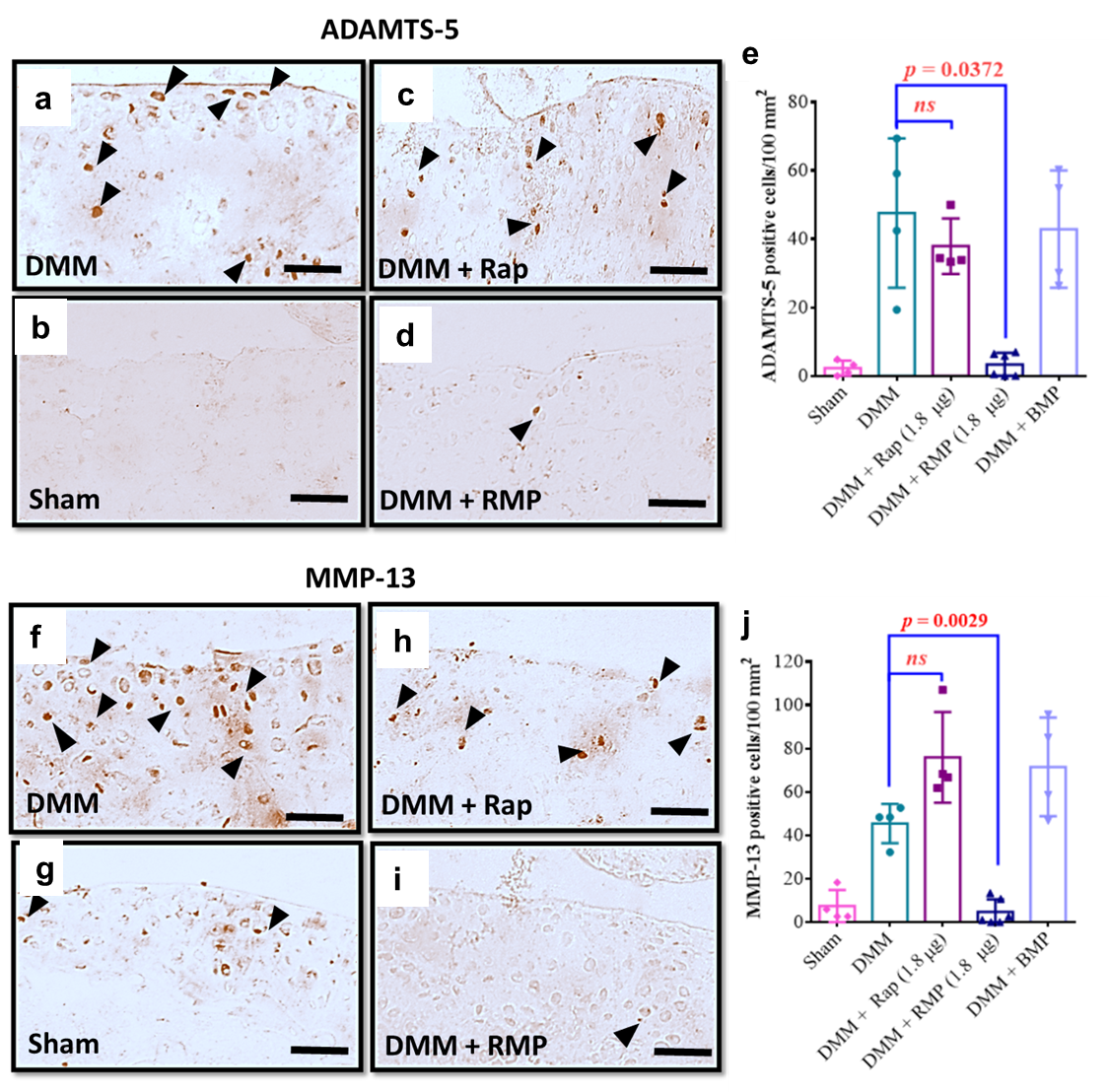
**

**Figure S10. RMPs in the prophylactic regimen reduced the markers of inflammation associated with OA in surgically induced murine OA models.** Immunohistochemical staining for ADAMTS-5 in **(a)** DMM, **(b)** Sham, **(c)** DMM operated with free rapamycin (1.8 µg) treatment and, **(d)** DMM operated with RMP (200 µg particles containing 1.8 µg rapamycin) treatment. **(e)** Quantification of ADAMTS-5 positive cells per 100 mm^2^. Immunohistochemical staining for MMP-13 in **(f)** DMM, **(g)** Sham, **(h)** DMM operated with rapamycin (1.8 µg) treatment and, **(i)** DMM operated with RMP (200 µg particles containing 1.8 µg rapamycin) treatment. **(j)** Quantification of MMP-13 positive cells per 100 mm^2^. The graphs represent the mean ± s.d. and *p* values were determined using one-way analysis of variance (ANOVA) or Kruskal Wallis test and Tukey’s post hoc analysis. DMM-Destabilization of Medial Meniscus model, BMP - Blank Microparticles, and RMP – Rapamycin Microparticles. *ns* - non significant; Scale bar, 50µm.


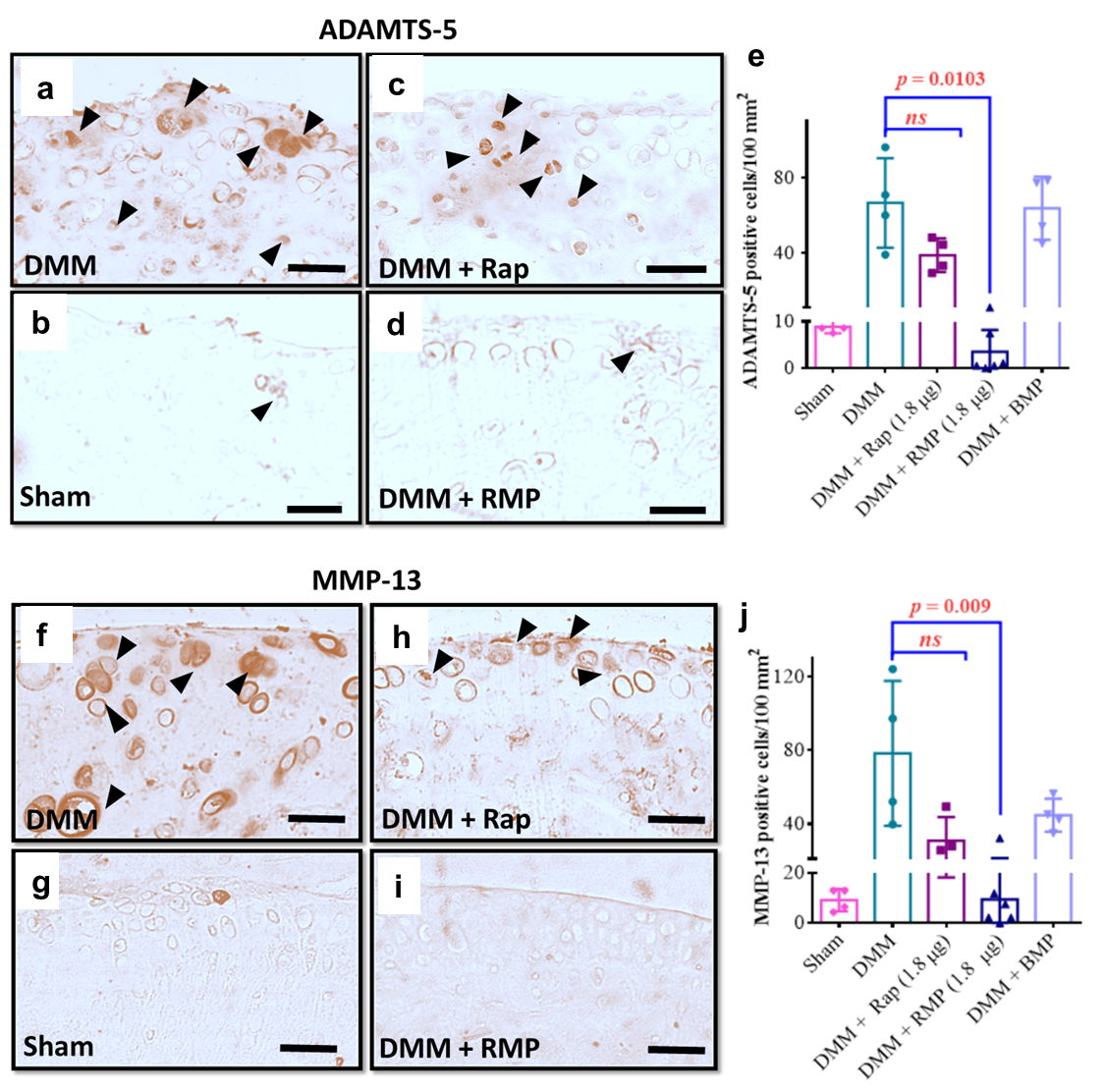


**Figure S11. RMPs in the therapeutic regimen reduced the markers of inflammation associated with OA in surgically induced murine OA models.** Immunohistochemical staining for ADAMTS-5 in **(a)** DMM, **(b)** Sham, **(c)** DMM operated with rapamycin (1.8 µg) treatment and, **(d)** DMM operated with RMP (200 µg particles containing 1.8 µg rapamycin) treatment. **(e)** Quantification of ADAMTS-5 positive cells per 100 mm^2^. Immunohistochemical staining for MMP-13 in **(f)** DMM, **(g)** Sham, **(h)** DMM operated with rapamycin (1.8 µg) treatment and, **(i)** DMM operated with RMP (200 µg particles containing 1.8 µg rapamycin) treatment. **(j)** Quantification of MMP-13 positive cells per 100 mm^2^. The graphs represent the mean ± s.d. and *p* values were determined using one-way analysis of variance (ANOVA) or Kruskal Wallis test and Tukey’s post hoc analysis. DMM-Destabilization of Medial Meniscus model, BMP - Blank Microparticles, and RMP – Rapamycin Microparticles. *ns* - non significant; Scale bar, 50µm.

**Table S1: PLGA microparticles of different molecular weights and PLA: PGA ratios, their respective sizes, and rapamycin encapsulation efficiency.**

| Molecular weight of PLGA  (Ratio of PLA: PGA) | Size measured by DLS [nm] | Rapamycin encapsulation efficiency [%] |
| --- | --- | --- |
| 10 kDa - 15 kDa (50:50) | 1039 ± 188 | 71.83 ± 4.889 |
| 75 kDa - 85 kDa (50:50) | 1153 ± 174.5 | 27.26 ± 2.704 |
| 190 kDa –240 kDa (85:15) | 1192 ± 232.2 | 51.10 ± 1.720 |

**Table S2: Details of volunteers who donated the knee joint samples following total knee arthroplasty.**

| Volunteer ID | Age/Gender | Primary Diagnosis |  |
| --- | --- | --- | --- |
| RMCH_001 | | 48/M | Osteoarthritis of knee |
| RMCH_002 | | 68/F | Osteoarthritis of knee |
| RMCH_003 | | 61/F | Osteoarthritis of knee |
